# SIRT3‐Mediated Deacetylation of DRP1^K711^ Prevents Mitochondrial Dysfunction in Parkinson's Disease

**DOI:** 10.1002/advs.202411235

**Published:** 2025-02-20

**Authors:** Ye Xi, Kai Tao, Xiaomin Wen, Dayun Feng, Zifan Mai, Hui Ding, Honghui Mao, Mingming Wang, Qian Yang, Jie Xiang, Jie Zhang, Shengxi Wu

**Affiliations:** ^1^ Department of Neurobiology School of Basic Medicine Fourth Military Medical University Xi'an Shaanxi 710032 China; ^2^ Department of Experimental Surgery Tangdu Hospital Fourth Military Medical University Xi'an Shaanxi 710038 China; ^3^ Department of Neurosurgery Tangdu Hospital Fourth Military Medical University Xi'an Shaanxi 710038 China; ^4^ Department of Biophysics Institute of Neuroscience NHC and CAMS Key Laboratory of Medical Neurobiology Zhejiang University School of Medicine Hangzhou 310058 China; ^5^ Institute of Neuroscience College of Medicine Xiamen University Xiamen Fujian 361105 China

**Keywords:** acetylation, DRP1^K711^, mitochondrial dysfunction, oxidative stress, Parkinson's disease, SIRT3

## Abstract

Dysregulation of mitochondrial dynamics is a key contributor to the pathogenesis of Parkinson's disease (PD). Aberrant mitochondrial fission induced by dynamin‐related protein 1 (DRP1) causes mitochondrial dysfunction in dopaminergic (DA) neurons. However, the mechanism of DRP1 activation and its role in PD progression remain unclear. In this study, Mass spectrometry analysis is performed and identified a significant increased DRP1 acetylation at lysine residue 711 (K711) in the mitochondria under oxidative stress. Enhanced DRP1^K711^ acetylation facilitated DRP1 oligomerization, thereby exacerbating mitochondrial fragmentation and compromising the mitochondrial function. DRP1^K711^ acetylation also affects mitochondrial DRP1 recruitment and fission independent of canonical S616 phosphorylation. Further analysis reveals the critical role of sirtuin (SIRT)‐3 in deacetylating DRP1^K711^, thereby regulating mitochondrial dynamics and function. SIRT3 agonists significantly inhibit DRP1^K711^ acetylation, rescue DA neuronal loss, and improve motor function in a PD mouse model. Conversely, selective knockout of SIRT3 in DA neurons exacerbates DRP1^K711^ acetylation, leading to increased DA neuronal damage, neuronal death, and worsened motor dysfunction. Notably, this study identifies a novel mechanism involving aberrant SIRT3‐mediated DRP1 acetylation at K711 as a key driver of mitochondrial dysfunction and DA neuronal death in PD, revealing a potential target for PD treatment.

## Graphical abstract

SIRT3‐mediated deacetylation of DRP1 at K711 resists mitochondrial dysfunction and DA neuronal death in PD, uncovering a treatment target.

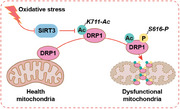

## 1 Introduction

Parkinson's disease (PD) is the second most prevalent neurodegenerative disorder globally,^[^ ^1^ ^]^ posing a serious threat to public health. Mitochondrial dysfunction‐induced dopaminergic (DA) neuronal death is a key hallmark of PD.^[^ ^2^, ^3^, ^4^, ^5^, ^6^ ^]^ However, the precise molecular pathways underlying mitochondrial dysfunction remain unclear. Mitochondria are dynamic organelles that undergo continuous fission and fusion to ensure proper mitochondrial biogenesis, functional homeostasis, and overall cellular viability.^[^ ^7^, ^8^ ^]^ Fusion compensates for mitochondrial defects by sharing components, whereas fission segregates the damaged mitochondria, thereby regulating morphology and trafficking to maintain the health of the mitochondrial network. Any disruption in this balance leads to oxidative stress, mitochondrial dysfunction, and metabolic alterations, ultimately promoting the development of mitochondrial‐related diseases.^[^ ^9^, ^10^ ^]^ Dysfunction of mitochondrial fission/fusion is closely associated with the onset of PD.^[^ ^11^, ^12^ ^]^ GTPase dynamin‐related protein 1 (DRP1), a major protein mediating mitochondrial division via GTP hydrolysis,^[^ ^10^ ^]^ is involved in the pathogenesis of various neurodegenerative diseases, such as Alzheimer's disease (AD), Amyotrophic lateral sclerosis, and Huntington's disease, including PD.^[^ ^13^, ^14^ ^]^ Neuronal stress, including oxidative stress that is widely observed in PD, induces various post‐translational modifications (PTMs), including phosphorylation, acetylation, ubiquitination, and ubiquitin‐like modifications, in DRP1.^[^ ^12^, ^15^, ^16^ ^]^ However, the specific PTMs of DRP1 that drive mitochondrial damage in DA neurons remain unknown.

Acetylation is the predominant PTM in mitochondrial proteins, with over 60% of mitochondrial proteins exhibiting acetylation at the modification sites.^[^ ^17^, ^18^, ^19^, ^20^ ^]^ Dysregulation of mitochondrial protein acetylation disrupts the oxidative metabolic pathways and compromises mitochondrial integrity and enzymatic activity, thereby damaging the DA neuronal function.^[^ ^21^, ^22^, ^23^, ^24^ ^]^ Uncovering the precise mechanism of DRP1 acetylation and its effect on mitochondrial function may reveal novel therapeutic targets for PD.

In this study, we identified a novel PTM of DRP1 at lysine 711 (K711) that was enhanced under oxidative stress. This modification promoted DRP1 recruitment to the mitochondria, inducing mitochondrial fission and altering the mitochondrial morphology and functions. Modulation of the acetylation level of K711 via SIRT3 improved mitochondrial health and reduced DA neuronal death. This study revealed the critical role of DRP1 acetylation in mitochondrial dysfunction and provided new insights into the pathological mechanisms of mitochondrial fission, thereby indicating novel therapeutic targets for PD.

## 2 Results

### 2.1 Oxidative Stress Induces DRP1 Hyperacetylation at K711

**Figure 1:**
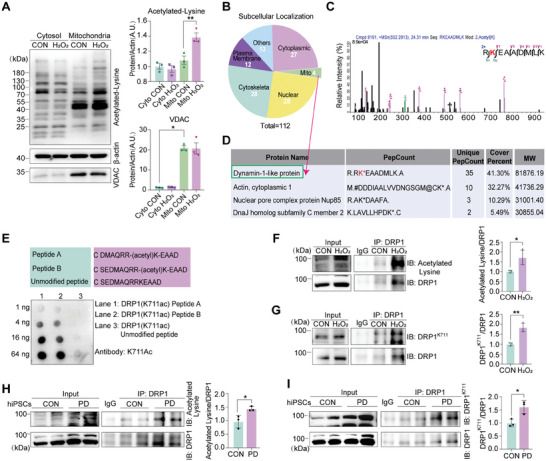
Enhanced K711 acetylation of DRP1 in mitochondria under oxidative stress. A) SH‐SY5Y cells were treated with 300 µM H_2_O_2_ for 18 h. Cytoplasmic and mitochondrial fractions were isolated and subjected to western blotting analysis to determine the acetylation levels in the indicated groups. Data are represented as the mean ± standard deviation (SD) (n = 3). *P < 0.05 and **P < 0.01 versus indicated group. B) Categorization of 112 acetylated proteins identified via mass spectrometry (MS) in H_2_O_2_‐treated SH‐SY5Y cells. C,D) MS analysis of K711 acetylation of DRP1 in the mitochondria and the list of the top four results based on pepcounts from mass spectrometry analysis. E) Antibody dot blot assay: Different doses of modified and unmodified peptides were immobilized onto a solid phase membrane and incubated with the antibody K711Ac, and binding between the peptides and antibody was analyzed. Dot blot results showed that the peptides exhibited over tenfold higher signal recognition for the modified peptide than for the unmodified peptide. F) SH‐SY5Y cells were treated with 300 µM H_2_O_2_ for 18 h. Acetylation level of DRP1 was measured via immunoprecipitation. Data are represented as the mean ± SD (n = 3). *P < 0.05 versus indicated group. G) SH‐SY5Y cells were treated with 300 µM H_2_O_2_ for 18 h. Analysis of DRP1^K711^ expression via immunoprecipitation. Data are represented as the mean ± SD (n = 3). **P < 0.01 versus indicated group. H) DRP1 acetylation level in PD patient‐derived hiPSCs via immunoprecipitation. Data are represented as the mean ± SD (n = 3). *P < *0.05* versus indicated group. I) DRP1^K711^ level in PD patient‐derived hiPSCs via immunoprecipitation. Data are represented as the mean ± SD (n = 3). *P < *0.05* and **P < *0.01* versus indicated group. Statistical analysis results are presented in Table S1 (Supporting Information).

Lysine is acetylated by transferring an acetyl group from acetyl coenzyme A to a lysine residue.^[^ ^25^ ^]^ This dynamic PTM occurs in both cytoplasmic and organellar proteins and influences various cellular processes including gene transcription and cell cycle progression.^[^ ^26^ ^]^ PTMs contribute to neuronal cell death. Mitochondrial dysfunction and morphological abnormalities are associated with various neurodegenerative diseases, including PD.^[^ ^14^, ^27^, ^28^, ^29^ ^]^ Therefore, modifications in mitochondrial acetylation may play a role in the pathogenesis of degenerative diseases. To systematically explore the acetylation modifications in mitochondrial proteins under oxidative stress conditions, cytoplasmic and mitochondrial proteins were isolated from SH‐SY5Y cells treated with hydrogen peroxide (H_2_O_2_) or a control vehicle. These proteins were subjected to western blotting using an acetylated lysine antibody. Western blotting results showed that a large amount of acetylable proteins were detected in the cytoplasm. However, from our experimental results, the changes in cytoplasmic acetylation after H_2_O_2_ treatment were not as significant as the changes in mitochondrial protein acetylation. Protein acetylation levels significantly increased in the mitochondria, but not in the cytoplasmic fraction, after H_2_O_2_ treatment (**Figure** **1**A). Voltage‐dependent anion channel was used as a mitochondrial marker.

To further explore the specific acetylation sites, proteins from H_2_O_2_‐treated SH‐SY5Y cells were subjected to an acetylome profiling assay (procedure described in detail in the Experimental Section). Our analysis revealed 112 acetylated proteins, including four mitochondrial proteins (Figure 1B). Among the acetylated proteins, a new lysine acetylation modification at residue K711 on DRP1, which exhibited the highest unique peptide count, was observed (Figure 1C,D). Then, we customized a specific antibody that could recognize DRP1^K711^ acetylation. We confirmed that this antibody could recognize the K711‐acylated DRP1 peptide (Figure 1E). Immunoprecipitation and western blotting clearly showed the marked upregulation of acetylation at lysine 711 (K711) of DRP1 under oxidative stress (Figure 1F,G). Concentration and time were determined based on the peak values of DRP1 protein expression, which increased after H_2_O_2_ treatment (Figure S1A–D, Supporting Information). These data suggest that oxidative stress induces elevated DRP1 acetylation at K711. Further, induced pluripotent stem cells (hiPSCs) originating from PD patients were utilized and differentiated into dopaminergic neurons (Figure S2, Supporting Information). Immunoprecipitation and Western blot analysis indicated an upregulated acetylation level of DRP1 and DRP1^K711^ in these neurons (Figure 1H,I). Importantly, these results were in line with cell experiments under oxidative stress conditions, thereby providing additional evidence suggesting that the acetylation levels of DRP1 and DRP1^K711^ in DA neurons of PD patients may be elevated.

### 2.2 Acetylation at K711 Promotes DRP1 Oligomerization, Leading to Mitochondrial Fission and Dysfunction

**Figure 2:**
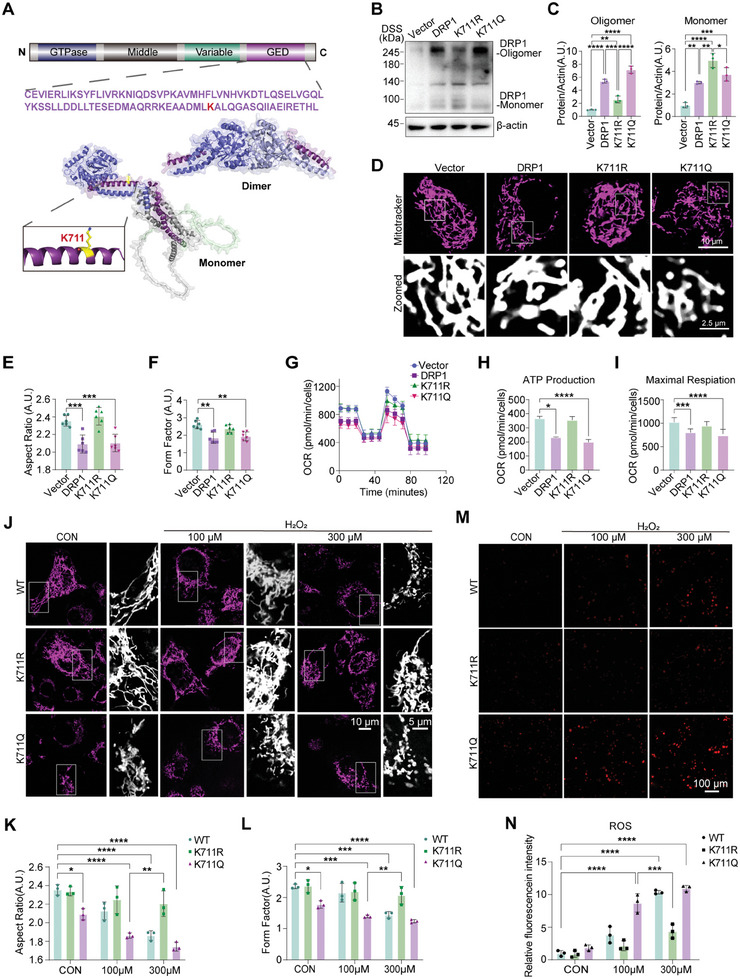
K711 acetylation of DRP1 alters the mitochondrial morphology and functions. A) Schematic representation of DRP1 structural domains, highlighting the K711 acetylation site. Monomeric and dimeric forms are shown. The monomeric and dimeric forms were simulated using software, based on the structures retrieved from the UniProt database. B,C) Western blotting analysis of DRP1 monomer and oligomer expression levels in HeLa cells transfected with the K711R and K711Q mutant plasmids. Data are represented as the mean ± SD (n = 3). *P < 0.05, **P < 0.01, ***P < 0.001, and ****P < 0.0001 versus indicated group. D–F) Mitochondrial morphology in HeLa cells transfected with the K711R and K711Q mutant plasmids was visualized via MitoTracker staining. The staining results were then analyzed to explore potential changes in mitochondrial shape and distribution caused by the K711R and K711Q mutant plasmids. **P < 0.01 and ***P < 0.001 versus indicated group. D) Representative images. E) Quantification of mitochondrial length and aspect ratio (AR, ratio between the major and minor axis of the ellipse equivalent to the mitochondrion). F) Quantification of mitochondrial form factor (FF, defined as [Pm2]/[4πAm], where Pm is the length of mitochondrial outline, and Am is the area of mitochondrion). Scale bars: 10 µm; 2.5 µm (zoomed regions). Data are represented as the mean ± SD (n = 6). **P < 0.01 and ***P < 0.001 versus indicated group. G–I) Oxygen consumption rate (OCR) analysis of ATP production levels and maximal respiratory capacity in HeLa cells transfected with the K711R and K711Q mutant plasmids. Data are represented as the mean ± SD (n = 3). *P < 0.05, ***P < 0.001 and ****P < 0.0001 versus indicated group. J–L) Mitochondrial morphology analysis of HeLa cells transfected with the K711R and K711Q mutant plasmids after treatment with 100 or 300 µM H_2_O_2_. J) Representative images. K) Quantification of mitochondrial AR. L) Quantification of mitochondrial FF. Scale bar: 10 µm. Data are represented as the mean ± SD (n = 3). *P < 0.05, **P < 0.01, ***P < 0.001, and ****P < 0.0001 versus indicated group. M,N) CellROX Deep Red staining of reactive oxygen species (ROS) generation in HeLa cells transfected with the K711R and K711Q mutant plasmids after treatment with 100 or 300 µM H_2_O_2_. M) Representative images. N) Quantification of ROS levels. Scale bar: 100 µm. Data are represented as the mean ± SD (n = 3). ***P < 0.001 and ****P < 0.0001 versus indicated group. Statistical analysis results are presented in Table S1 (Supporting Information).

DRP1 typically exists as a homotetramer in the cytoplasm and forms high‐order structures in the outer mitochondrial membrane.^[^ ^30^ ^]^ DRP1 contains four distinct domains: N‐terminal GTPase domain (GTP), middle domain, variable domain, and C‐terminal GTPase effector domain (GED)^[^ ^16^, ^31^ ^]^ (**Figure** **2**A). We constructed the structural diagram based on the structural information obtained from the UniProt database. C‐terminal GED is critical for GTPase activity and mitochondrial fission.^[^ ^32^ ^]^ In the field of biomolecules, the modification of phosphorylation, methylation, and acetylation sites in the GED domain may be mediated by changing enzyme activity, protein–protein interaction, and subcellular localization, causing a change in its function. Here, acetylation residue K711 was in GED, suggesting that this acetylation modification affects DRP1 oligomerization (Figure 2A).

According to the MS results, we established the classic mutations for deacetylation (K mutated to R) and acetylation mimicry (K mutated to Q)^[^ ^33^, ^34^, ^35^ ^]^ to test our hypothesis. Subsequently, we generated the acetylated (K711Q) and deacetylated (K711R) DRP1 vectors and transfected them, in combination with the wild‐type DRP1 vector, into HeLa cells. Initially, we demonstrated that following mutation at the K711 site, it failed to be recognized by the acetylated antibody specific to 711, thereby confirming this site as an acetylated site (Figure S3A–C, Supporting Information). Upon cross‐linking with Disuccinimidyl suberate (DSS), wild‐type DRP1 exhibited enhanced oligomerization, which was further amplified by the acetylation‐mimicking K711Q mutation. In contrast, deacetylated K711R mutation resulted in the formation of monomers (Figure 2B,C). Furthermore, overexpression of the wild‐type and K711Q mutants, but not K711R DRP1, promoted the fragmented mitochondrial morphology (Figure 2D–F) and significantly decreased the membrane potential compared to those in the controls (Figure S4A,B, Supporting Information). Along with the morphological changes in the mitochondria, ATP production and maximal respiratory capacity were reduced in the wild‐type and K711Q DRP1‐expressing cells, but not in K711R DRP1‐expressing cells (Figure 2G–I). These findings suggest that the simulated acetylation K711Q mutation activates DRP1, leading to mitochondrial fragmentation and dysfunction.

Under oxidative stress, in addition to observing the results consistent with those previously obtained, namely that the overexpression of the wild‐type and K711Q mutants, but not K711R DRP1, promoted the fragmented mitochondrial morphology (Figure 2J–L) and significantly decreased the membrane potential compared to those in the controls (Figure S4C,D, Supporting Information), we also found that up to 300 µm H_2_O_2_ treatment induced significant mitochondrial fission in wild‐type DRP1‐expressing HeLa cells. Notably, 100 µm H_2_O_2_ induced comparable mitochondrial fission in K711Q‐DRP1‐expressing cells. Conversely, overexpression of K711R‐DRP1 almost prevented H_2_O_2_‐induced mitochondrial fragmentation (Figure 2J–L). Tetramethylrhodamine ethyl ester (TMRE) staining further revealed that K711Q DRP1 mutation accelerated the membrane potential decline induced by H_2_O_2_ treatment, whereas K711R DRP1 mutation abolished this effect induced by H_2_O_2_ even at 300 µm (Figure S4C,D, Supporting Information). Simultaneously, K711Q led to an upward trend in ROS production compared with the control group. Despite exacerbating neuronal damage via increased reactive oxygen species (ROS) production due to mitochondrial dysfunction, K711R mutation inhibited H_2_O_2_‐induced mitochondrial ROS generation (Figure 2M,N). These findings suggest that K711 acetylation induces mitochondrial damage, leading to increased ROS production under mild oxidative stress. Conversely, deacetylation at K711 exerts protective effects on mitochondrial morphology and function, reducing ROS production even under high oxidative stress.

Taken together, our data indicate that acetylation at K711 promotes DRP1 oligomerization, leading to mitochondrial fission and dysfunction.

### 2.3 Deacetylation at K711 of DRP1 Abolishes the Effects of Serine 616 (S616) Phosphorylation

**Figure 3:**
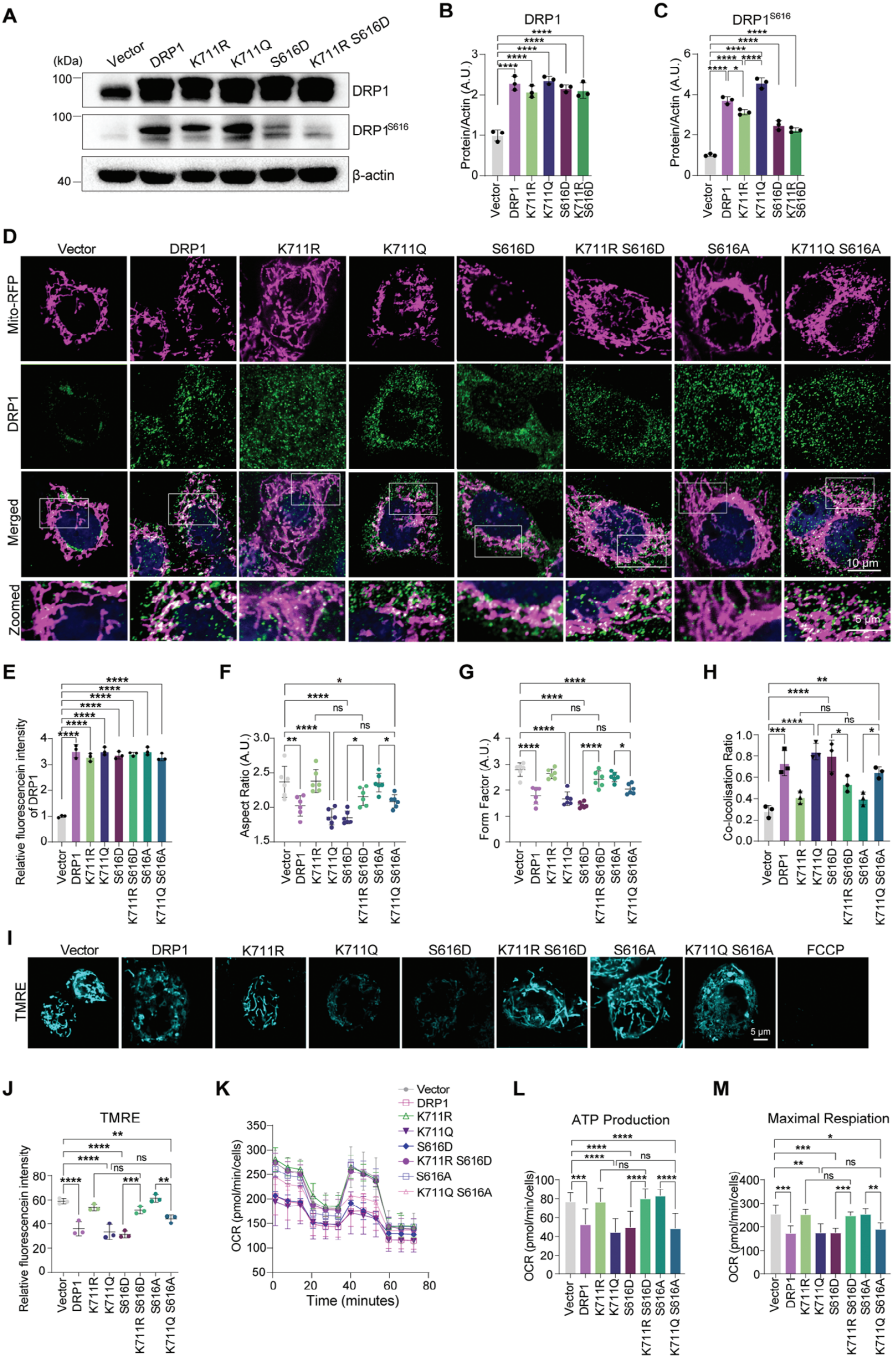
K711 acetylation of DRP1 regulates the mitochondrial morphology and functions synergistically with S616‐induced fission. A–C) Western blotting analysis of DRP1 and phosphorylated DRP1 (S616) levels in HeLa cells transfected with plasmids encoding wild‐type DRP1, K711R, K711Q, S616D and K711R S616D. Data are represented as the mean ± SD (n = 3). *P < 0.05 and ****P < 0.0001 versus indicated group; *ns*, not significant. D–H) Immunofluorescence microscopy showing the effects of distinct DRP1 variants (wild‐type DRP1, K711R, K711Q, S616D, K711R S616D, S616A, and K711Q S616A) on mitochondrial morphology (visualized using Mito‐RFP), DRP1 levels and the co‐labeling of DRP1 and mitochondria in HeLa cells. D) Representative images. E) Quantification of DRP1 expression levels. F) Quantification of the morphological index AR of mitochondrial. G) Quantification of the morphological index FF of mitochondria. H) Quantification of DRP1 co‐localization in mitochondria. Scale bar: 10 µm. Data are represented as the mean ± SD (n = 3–6). *P < 0.05, **P < 0.01, ***P < 0.001 and ****P < 0.0001 versus indicated group; *ns*, not significant. I,J) Mitochondrial membrane potential (ΔΨm) in HeLa cells expressing DRP1 variants assessed via TMRE staining. ** P < 0.01, ***P < 0.001 and ****P < 0.0001 versus indicated group; *ns*, not significant. K–M) Oxygen consumption rate (OCR) analysis of the effects of distinct DRP1 variants on ATP production and maximal respiratory capacity in HeLa cells. Data are represented as the mean ± SD (n = 4). *P < 0.05, **P < 0.01, ***P < 0.001, and ****P < 0.0001 versus indicated group; *ns*, not significant. Statistical analysis results are presented in Table S1 (Supporting Information).

Phosphorylation of DRP1 at S616 promotes oligomerization and induces mitochondrial fission.^[^ ^36^, ^37^ ^]^ Here, the deacetylated K711R mutation significantly prevented S616 phosphorylation in DRP1 compared to that in wild‐type and K711Q DRP1; meanwhile, the phosphorylation expression of S616 in the mutated S616 group was also decreased, possibly due to the inability of the phosphorylation antibody to recognize the site after mutation (**Figure** **3**A–C). Considering the roles of K711 acetylation and S616 phosphorylation in promoting mitochondrial fission, we investigated their combined effects on DRP1 functions. We generated two types of DRP1 mutants, namely S616D and S616A. Specifically, we introduced the K711R mutation along with a phosphorylation‐mimic mutation at S616, resulting in the mutant K711R S616D; and we also introduced the K711Q mutation accompanied by a phosphorylation‐deficient mutation at S616, generating the mutant K711Q S616A. DRP1 immunostaining confirmed that the expression levels of DRP1 were comparable among the wild‐type, K711R, K711Q, S616D, K711R S616D, S616A and K711Q S616A DRP1‐transfected cells (Figure 3D,E). Acetylation mimicking the mutation of DRP1 (K711Q) promoted mitochondrial fission; this effect was not influenced by the phosphorylation‐dead mutation at S616 (K711Q S616A). Moreover, the mitochondrial fragmentation morphology in the K711Q S616A group was more significant than that in the S616A group. Similarly, resistance to mitochondrial‐fragmentation by K711R‐mutated DRP1 was not affected by another phosphorylation mimic at S616 (K711R S616D). However, the mitochondrial elongated form in the K711R S616D mutant group was more obvious than that in the S616D group (Figure 3F,G). Furthermore, a pronounced divergence in the co‐expression index of Mito‐RFP and DRP1 was observed in DRP1‐, K711Q‐, S616D‐, and K711Q S616A‐transfected cells relative to that in the controls. This observation indicated increased DRP1 translocation to the mitochondria (Figure 3H). TMRE staining revealed a significant decrease in the mitochondrial membrane potential in cells transfected with the acetylation mimic DRP1 mutation (K711Q), and this effect was not influenced by the phosphorylation‐dead mutation at S616 (K711Q S616A). While the mitochondrial membrane potential in the S616D mutant group was significantly decreased compared to that in the K711R S616D group. Similarly, the protective effect against changes in membrane potential exerted by K711R mutation was not affected by the phosphorylation‐mimicking mutation at S616 (K711R S616D). While the mitochondrial membrane potential in the K711Q S616A mutant group was significantly decreased compared to that in the S616A group (Figure 3I,J). Seahorse oxygen consumption rate (OCR) assays revealed that the decline in maximal respiratory capacity and ATP production in K711Q‐ and K711Q S616A‐transfected cells was not influenced by the S616 mutation, with the K711R‐ and no significant differences observed between the K711R S616D‐transfected cells and controls (Figure 3K–M). These results suggest that K711 acetylation of DRP1 modulates mitochondrial morphology and function independent of S616 phosphorylation. The K711R mutation could potentially lead to conformational changes in the protein, affecting kinase binding and phosphorylation site accessibility, as with potential disruptions to phosphorylation of adjacent residues like S616. Regarding subcellular localization, as seen in Figure 3D,H, the K711R mutation impact the translocation of DRP1 to mitochondria, which might in turn affect the phosphorylation process. However, the exact mechanism underlying these phenomena requires further investigation.

### 2.4 SIRT3 Modulates Mitochondrial Functions and Morphology by Regulating DRP1^K711^ Acetylation

Lysine acetylation is regulated by the SIRT family, which are NAD+‐dependent histone deacetylases.^[^ ^38^ ^]^ SIRTs contain seven members (SIRT1–7). SIRT3 and SIRT5 are in the mitochondria and play critical roles in regulating mitochondrial function.^[^ ^39^, ^40^ ^]^ To identify the specific SIRT mediating the regulation of DRP1 acetylation at K711, HeLa cells were transfected with small interfering RNAs (siRNAs) targeting SIRT3 and SIRT5. The knockdown efficacy of these siRNAs was confirmed via western blotting (Figure S5A–D, Supporting Information). We found that knockdown *SIRT3*, but not *SIRT5*, induced significant mitochondrial fragmentation and increased the co‐localization index of mitochondria and acetylation levels in HeLa cells compared to those in the controls (Figure S6A–D, Supporting Information). To further confirm this result, HeLa cells were treated with the SIRT3 inhibitor, 3‐[(4‐chlorophenyl)amino]methyl]‐5‐(2‐pyridinyl)isoxazole (3‐TYP),^[^ ^41^ ^]^ and SIRT5 inhibitor, suramin.^[^ ^42^ ^]^ Consistent with the siRNA data, treatment with 3‐TYP, but not suramin, induced mitochondrial fragmentation and increased the co‐localization of mitochondria and acetylation (Figure S6E–H, Supporting Information). Additionally, mitochondrial membrane potential was significantly reduced by genetic or pharmacological inhibition of SIRT3 (Figure S6I–L, Supporting Information). Seahorse analysis demonstrated a significant reduction in maximal respiratory capacity and ATP production in cells treated with siSIRT3 or 3‐TYP (Figure S6M–P, Supporting Information). Additionally, knockout of *SIRT3*, but not *SIRT5*, significantly elevated K711 acetylation (Figure S6Q–S, Supporting Information). Collectively, these results suggest SIRT3 as a key regulator of DRP1 deacetylation, whose inhibition leads to mitochondrial fragmentation and dysfunction.

**Figure 4:**
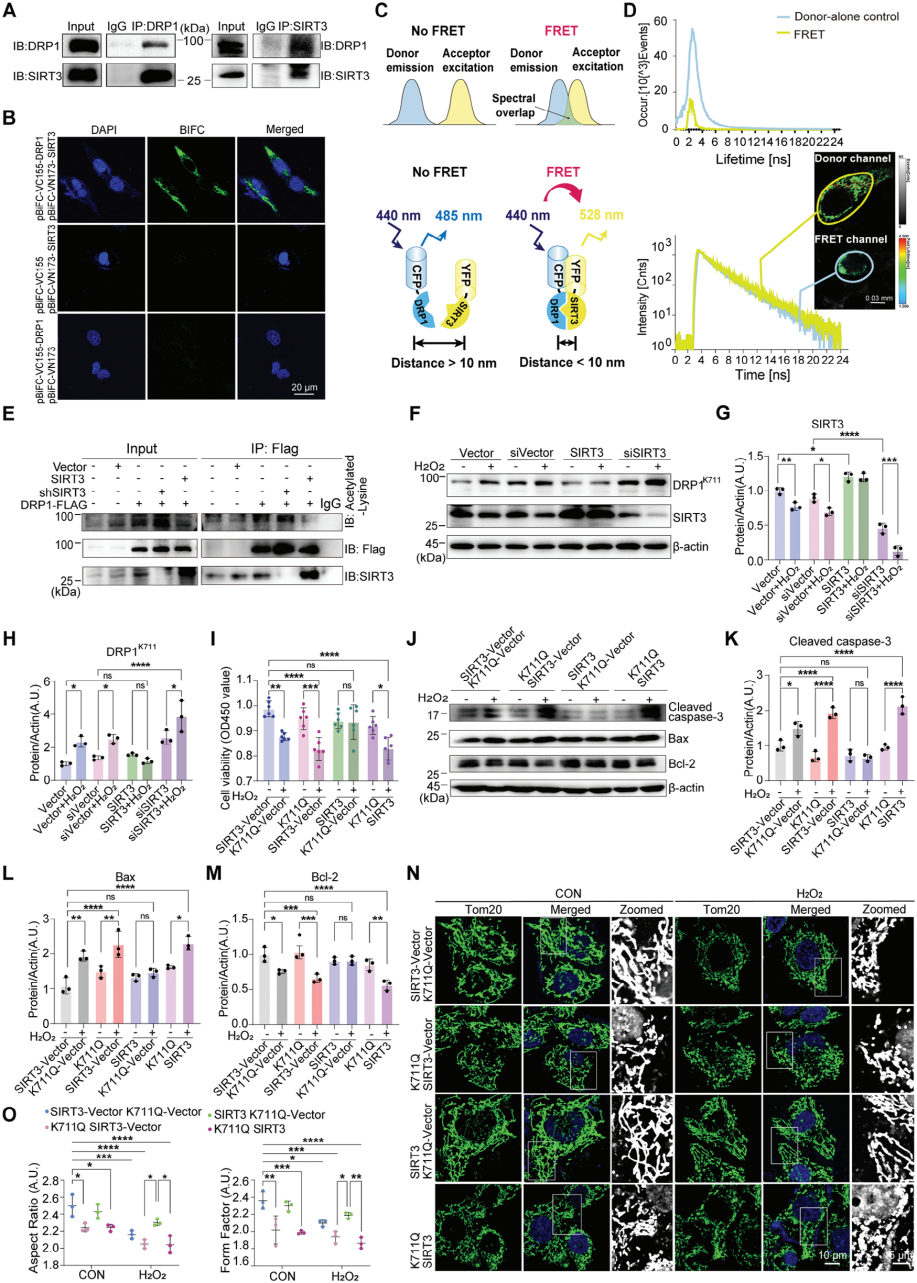
SIRT‐3 regulates mitochondrial function and morphology via K711 acetylation of DRP1. A) Co‐immunoprecipitation assay showing the interaction between DRP1 and SIRT3 in HeLa cells. B) Bimolecular fluorescence complementation (BiFC) assay confirmed the interaction between DRP1 and SIRT3 in HeLa cells. Fusion constructs of DRP1‐HA and SIRT3‐FLAG were co‐expressed, and green fluorescence indicates protein interaction. Scale bar: 20 µm. C) The schematic diagram of the FLIM – FRET experiment principle for CFP – DRP1 and YFP – SIRT3. Representative images showing the lifetime distribution. D)Time‐correlated single‐photon counting‐fluorescence lifetime imaging microscopy (TCSPC FLIM) with Förster resonance energy transfer (FRET) further confirmed the interaction between DRP1 and SIRT3. Comparison of the decay data and fitting curves of cells expressing CFP alone and those co‐expressing CFP and EYFP confirmed FRET, indicating the interaction between DRP1 and SIRT3. Scale bar: 0.03 mm. E) Co‐immunoprecipitation experiments using shSIRT3 or SIRT3‐overexpressing cell lines co‐transfected with DRP1‐Flag revealed the regulatory role of SIRT3 in DRP1 acetylation. F–H) Western blotting analysis showing the impact of SIRT3 overexpression or knockdown on the acetylation levels of DRP1 at K711 in HeLa cells under the condition of H₂O₂‐induced oxidative stress. Data are represented as the mean ± SD (n = 3). *P < 0.05, **P < 0.01, ***P < 0.001, and ****P < 0.0001 versus indicated group; *ns*, not significant. I) After being co‐transfected with the SIRT3 and K711Q plasmids, the CCK‐8 assay was conducted to show the protective effects of SIRT3 against H₂O₂‐induced cell death in HeLa cells. Data are represented as the mean ± SD (n = 6). *P < 0.05, **P < 0.01, ***P < 0.001, and ****P < 0.0001 versus indicated group; *ns*, not significant. J–M) After being co‐transfected with the SIRT3 and K711Q plasmids and subsequently treated with H₂O₂, Western blotting analysis was performed to detect the expression levels of cleaved caspase‐3, Bax, and Bcl‐2 in HeLa cells, which confirmed the protective effects of SIRT3 against H₂O₂‐induced apoptosis in HeLa cells. Data are represented as the mean ± SD (n = 3). *P < 0.05, **P < 0.01, ***P < 0.001, and ****P < 0.0001 versus indicated group; *ns*, not significant. N,O) Immunofluorescence staining with Tom20 antibody revealed the impact of SIRT3 on mitochondrial morphology in HeLa cells. Quantification of mitochondrial AR and FF is shown. Scale bar: 10 µm. Data are represented as the mean ± SD (n = 3). *P < 0.05, **P < 0.01, ***P < 0.001, and ****P < 0.0001 versus indicated group; *ns*, not significant. Statistical analysis results are presented in Table S1 (Supporting Information).

To determine whether SIRT3 physically interacts with DRP1, we performed co‐immunoprecipitation (Co‐IP), bimolecular fluorescence complementation (BiFC), fluorescence lifetime imaging microscopy‐Förster resonance energy transfer (FLIM‐FRET) assays and HIS – Pulldown Recombinant Protein Interaction Assay to investigate their interactions in vitro and in vivo. Co‐IP experiments revealed the interaction between DRP1 and SIRT3 (**Figure** **4**A). Using BiFC, DRP1‐HA, and SIRT3‐FLAG were fused to the pBiFC‐VC155 and pBiFC‐VN173 vectors, respectively. GFP fluorescent signals indicated their interaction in the cytoplasm in vivo (Figure 4B; Figure S7A, Supporting Information). Using plasmids fusing DRP1 with a cyan fluorescent protein (CFP) and SIRT3 with an enhanced yellow fluorescent protein (EYFP), FLIM‐FRET experiments revealed a decreased fluorescence lifetime, indicating the interaction between DRP1 and SIRT3 within 10 nm (Figure 4C,D). Using HIS magnetic beads to pull down the HIS‐tagged DRP1 recombinant protein that interacted with the SIRT3 recombinant protein. The interaction between the two proteins was then verified by Coomassie Brilliant Blue staining, which clearly demonstrated the presence of both proteins in the pulled‐down complex, providing further evidence to support their interaction (Figure S7B, Supporting Information). Immunoprecipitation and western blotting were used to assess the acetylation of DRP1 under SIRT3 knockdown and overexpression. The acetylation of DRP1 was found to be increased upon *SIRT3* knockdown, and the acetylation of DRP1 decreased when *SIRT3* was overexpressed (Figure 4E). *SIRT3* knockdown enhanced H₂O₂‐induced acetylation at DRP1^K711^, whereas SIRT3 overexpression prevented this effect (Figure 4F–H). To assess the effects of SIRT3‐mediated regulation of K711 acetylation on cell survival and apoptosis, HeLa cells co‐transfected with SIRT3 and K711Q‐DRP1 plasmids were subjected to cell counting kit‐8 assays, and apoptotic marker protein levels were determined via western blotting. SIRT3 overexpression protected against the decrease in cell viability caused by oxidative stress; however, the viability of K711Q‐transfected cells overexpressing SIRT3 remained significantly decreased after oxidative stress treatment (Figure 4I). Consistent with these findings, K711Q‐DRP1 overexpression exacerbated H₂O₂‐induced apoptosis, whereas SIRT3 overexpression reversed this effect. However, SIRT3 overexpression failed to reverse the K711Q‐exacerbated H₂O₂‐induced apoptosis (Figure 4J–M).

Immunofluorescence staining with the Tom20 antibody was used to evaluate mitochondrial fragmentation (Figure 4N). SIRT3 overexpression protected against H₂O₂‐induced fragmentation, but this effect was lost in the presence of the K711Q mutation (Figure 4N,O). SIRT3 overexpression mitigated the H₂O₂‐induced decline in mitochondrial membrane potential as revealed by membrane potential staining. Conversely, the K711Q mutation exacerbated this decline, which was not reversed by SIRT3 (Figure S8A,B, Supporting Information). These findings suggest that after mutating the K711 site, SIRT3 loses its ability to regulate acetylation at this site to improve mitochondrial morphology and function.

### 2.5 SIRT3 Protects against 1‐methyl‐4‐phenylpyridinium (MPP^+^)‐Induced Apoptosis by Regulating DRP1^K711^ Acetylation In Vitro

**Figure 5:**
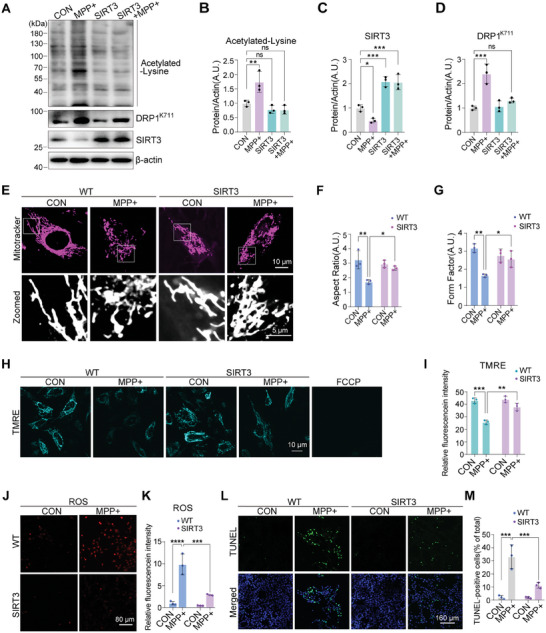
SIRT3 protects against MPP^+^‐induced apoptosis by regulating DRP1^K711^ acetylation. A–D) Western blotting analysis of the effects of SIRT3 overexpression on acetylated lysine, DRP1^K711^, and SIRT3 levels in SH‐SY5Y cells after MPP^+^ treatment. Data are represented as the mean ± SD (n = 3). *P < 0.05, **P < 0.01, and ***P < 0.001 versus indicated group; *ns*, not significant. E–G) Mitochondrial morphology in SH‐SY5Y cells after MPP^+^ treatment was assessed via MitoTracker staining. Quantification of mitochondrial AR and FF is shown. Scale bars: 10 µm; 5 µm (zoomed). Data are represented as the mean ± SD (n = 3). *P < 0.05 and **P < 0.01 versus indicated group; *ns*, not significant. H,I) Mitochondrial membrane potential (ΔΨm) in SH‐SY5Y cells after MPP+ treatment evaluated via TMRE staining. Scale bar: 10 µm. Data are represented as the mean ± SD (n = 3). **P < 0.01 and ***P < 0.001 versus indicated group. J,K) ROS generation in MPP+ treated SH‐SY5Y cells assessed via CellROX Deep Red staining. Scale bar: 80 µm. Data are represented as the mean ± SD (n = 3). ***P < 0.001 and ****P < 0.0001 versus indicated group. L) TUNEL analysis of MPP+ treated SH‐SY5Y cell apoptosis. Scale bar: 160 µm. M) Quantification of TUNEL‐positive cells. Data are represented as the mean ± SD (n = 3). ***P < 0.001 versus indicated group. Statistical analysis results are presented in Table S1 (Supporting Information).

SIRT3 protected mitochondrial morphology and function by regulating DRP1^K711^ acetylation. We further investigated whether this regulation is observed in oxidative stress‐induced PD cell and mouse models. SH‐SY5Y cells were treated with MPP^+^, which is a neurotoxin that enters dopaminergic neurons, disrupts mitochondrial function, and causes cell death. MPP^+^ significantly increased the levels of total acetylated lysine in a time‐ and dose‐dependent manner (Figure S9A–D, Supporting Information). Moreover, the total acetylation level and specific K711 acetylation of DRP1 were increased by MPP^+^ treatment, which was reversed by SIRT3 overexpression (**Figure** **5**A–D). Treatment with the SIRT3 inhibitor, 3‐TYP, accelerated MPP^+^‐induced DRP1 acetylation (Figure S10A–D, Supporting Information).

Mitochondrial fragmentation induced by MPP^+^ was effectively blocked by SIRT3 overexpression (Figure 5E–G). Furthermore, cells treated with MPP^+^ exhibited significantly decreased mitochondrial membrane potential, but this effect was reversed by SIRT3 overexpression (Figure 5H,I). Moreover, 3‐TYP exacerbated both mitochondrial fragmentation and a decrease in mitochondrial membrane potential induced by MPP^+^ administration (Figure S10E–I, Supporting Information). Finally, we assessed the ROS levels and apoptosis in these cells. MPP^+^ treatment significantly increased the ROS levels, but this effect was reversed by SIRT3 overexpression (Figure 5J,K). TdT‐mediated dUTP nick‐end labeling (TUNEL) staining confirmed that SIRT3 overexpression effectively protected against MPP^+^‐induced apoptosis (Figure 5L,M).

These findings suggest that SIRT3 protects against MPP^+^‐induced apoptosis by regulating DRP1^K711^ acetylation, thereby preserving the mitochondrial morphology and function and reducing ROS production.

### 2.6 DRP1^K711^ Acetylation‐Induced Mitochondrial, Neuronal and Motor Deficits in Mice

**Figure 6:**
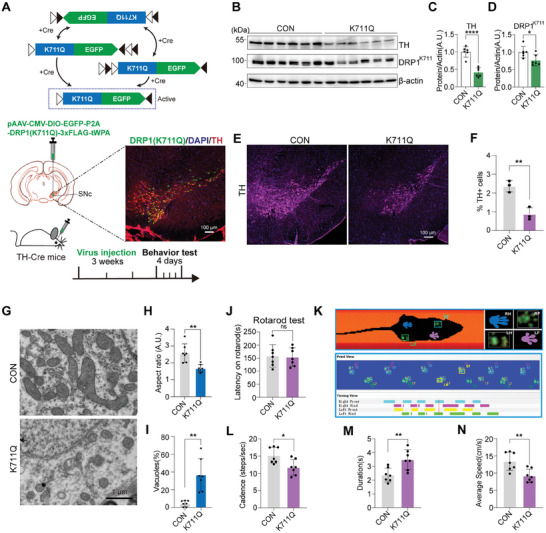
DRP1^K711^ acetylation induces mitochondrial and neuronal damage with motor deficits in mice. A) Illustration of generating TH‐specific DRP1^K711Q^ overexpression mice through stereotaxic virus injection into the SNc of TH‐cre mice. Immunofluorescence staining images were presented to show the injection site for confirmation of the targeted area. Scale bars: 100 µm. B–D) Western blotting analysis of TH and DRP1^K711^ levels in the SNc brain tissues of DRP1^K711Q^ overexpression mice. Data are represented as the mean ± SD (n = 6). *P < 0.05 and ****P < 0.0001 versus indicated group; *ns*, not significant. E,F) Immunofluorescence staining and analysis of TH expression levels in the SNc region of DRP1^K711Q^ overexpression mice. Data are represented as the mean ± SD (n = 3). **P < 0.01 versus indicated group. Scale bars: 100 µm. G–I) Electron microscopy analysis of mitochondria morphology in the SNc brain tissues of DRP1^K711Q^ overexpression mice. Quantification of mitochondrial AR and Vacuoles% is shown. Data are represented as the mean ± SD (n = 3). **P < 0.01 versus indicated group. Scale bars: 1 µm. J–N) Motor behavioral tests of DRP1^K711Q^ overexpression mice. J) Rotarod test analysis of motor function. Data are represented as the mean ± SD (n = 7). *ns*, not significant. K) Test diagram from the Catwalk system. The system shows green paw prints and records parameters after recognition. L–N) Catwalk gait analysis of cadence, duration, and average speed. Data are represented as the mean ± SD (n = 7). *P < 0.05 and **P < 0.01 versus indicated group.

To validate the critical role of K711 site acetylation within the dopaminergic neurons of the substantia nigra pars compacta (SNc) in animals, we generated a transgenic mouse model overexpressing K711Q specifically in the SNc region. This was achieved by stereotactic injection of pAAV‐CMV‐DIO‐EGFP‐P2A‐DRP1 (K711Q)‐3 × FLAG‐tWPA into the SNc of TH‐cre mice (**Figure** **6A**). Western blotting demonstrated a significant reduction in both DRP1^K711^ and TH protein levels (Figure 6B–D). Immunohistochemical analysis staining revealed a notable decline in the expression of TH‐positive neurons in the SNc tissues of the K711Q overexpression mice (Figure 6E,F). Electron microscopy examination revealed that the mitochondria of the SNc neurons in the K711Q overexpression group tended to have a more rounded and fragmented morphology, and the count of vacuoles within the mitochondria significantly increased, indicating mitochondrial damage (Figure 6G–I).

In the motor behavior tests, no significant difference was found between the K711Q overexpression mice and the control group in the rotarod test (Figure 6J). However, in the gait test, the cadence and average speed of the K711Q overexpression mice were significantly decreased and the platform time was increased compared with the control group (Figure 6K–N). Overall, these results suggest that K711 acetylation can impair mitochondrial morphology and DA neurons, leading to impaired motor function in animals to a certain extent.

### 2.7 SIRT3 Activation Suppresses DA Neuronal Loss and Motor Dysfunction by Inhibiting DRP1^K711^ Acetylation in 1‐methyl‐4‐phenyl‐1,2,3,6‐tetrahydropyridine (MPTP)‐Treated Mice

**Figure 7:**
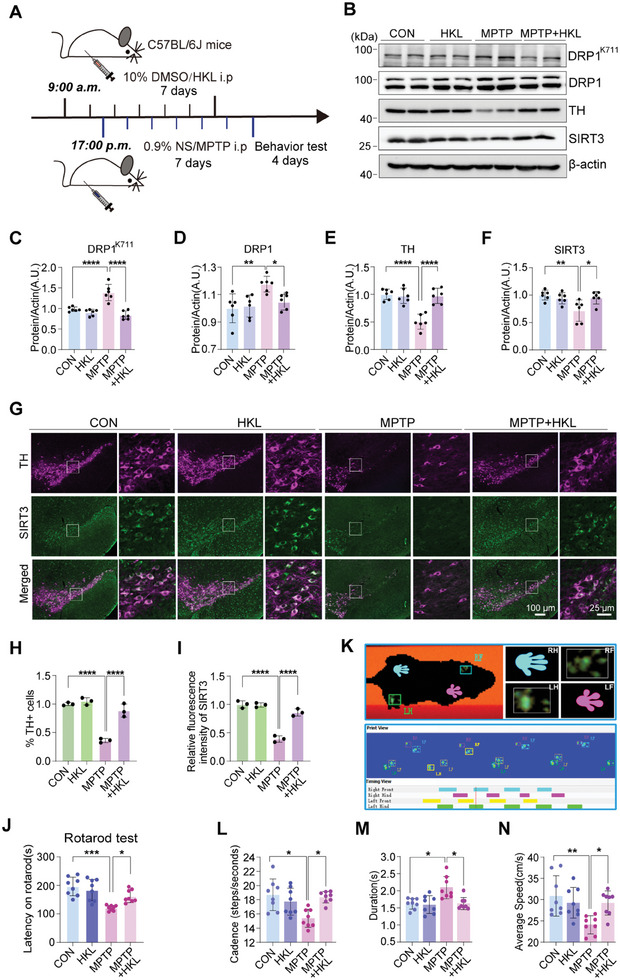
SIRT3 agonist HKL protects dopaminergic neurons and alleviates motor dysfunction in the MPTP‐induced PD mouse model by modulating DRP1 acetylation. A) Experimental timeline for the administration of HKL and MPTP to establish a PD mouse model and subsequent behavioral testing. B–F) Western blotting analysis of DRP1^K711^, DRP1, TH, and SIRT3 levels in SNc brain tissue samples of MPTP‐induced PD model mice treated with the SIRT3 agonist, HKL. Data are represented as the mean ± SD (n = 6). *P < 0.05, **P < 0.01, and ****P < 0.0001 versus indicated group. G–I) Immunofluorescence staining and analysis of TH and SIRT3 expression levels in the SNc region of HKL‐treated MPTP‐induced C57BL/6J PD model mice. Data are represented as the mean ± SD (n = 3). ****P < 0.0001 versus indicated group. J) Rotarod test analysis of motor function in MPTP‐induced PD mice treated with HKL. Data are represented as the mean ± SD (n = 8). *P < 0.05 and ***P < 0.001 versus indicated group. K) Test diagram from the Catwalk system. The system shows green paw prints and records parameters after recognition. L–N) Catwalk gait analysis of cadence, duration, and average speed in HKL‐treated MPTP‐induced PD mice. Data are represented as the mean ± SD (n = 8). *P < 0.05 and **P < 0.01 versus indicated group.

To investigate the neuroprotective effects of SIRT3 in vivo, we used MPTP which is a lipid – soluble compound that can cross the blood–brain barrier and is metabolized in the brain to the toxic MPP⁺ to establish a subacute PD mouse model (**Figure** **7**A) and 6‐hydroxydopamine (6‐OHDA) to establish an acute PD mouse model (Figure S11A, Supporting Information). Mice were treated with the SIRT3 agonist, honokiol (HKL), for seven days. The substantia nigra pars compacta (SNc) tissues were isolated for western blotting. Based on our preliminary experiments, we observed that excessive doses of HKL could trigger mortality in animals within the MPTP group. Consequently, the presently selected optimal dose operates under normal physiological conditions without significantly reducing the DRP1 acetylation level of mice. MPTP treatment significantly increased the DRP1 protein and K711 acetylation levels. However, these effects were reversed by the SIRT3 agonist, HKL. Additionally, HKL reversed the MPTP‐induced decrease in SIRT3^[^ ^43^ ^]^ and tyrosine hydroxylase (TH) levels (Figure 7B–F). Consistent with the findings in the MPTP‐induced model, HKL increased the SIRT3 levels and decreased 6‐OHDA‐induced acetylation of lysine 711 in DRP1. A decrease in TH‐positive neurons induced by 6‐OHDA was also inhibited by HKL (Figure S11B–E, Supporting Information). Immunohistochemical analysis revealed that the SIRT3 agonist ameliorated the MPTP‐induced loss of DA neurons and decreased SIRT3 expression in SNc brain tissues (Figure 7G–I). Notably, staining results in the 6‐OHDA model were consistent with those in the MPTP model (Figure S11F–H, Supporting Information).

Next, we conducted rotarod and gait tests to evaluate the effects of SIRT3‐mediated neuroprotection on motor behaviors. Behavioral analyses indicated that MPTP‐induced PD mice exhibited significantly decreased latency to fall in the rotarod test, which was reversed by HKL (Figure 7J). HKL significantly attenuated MPTP‐induced gait impairment, including reduced cadence and average speed and increased duration (Figure 7K–N). These results highlight the significant improvement in behavioral impairments in MPTP‐induced PD model mice after treatment with the SIRT3 agonist. Additionally, HKL improved the behavioral impairment in the 6‐OHDA‐induced acute PD model mice (Figure S11I–L, Supporting Information).

### 2.8 SIRT3 Deficiency Exacerbates DA Neuron Loss and Motor Dysfunction in PD Model Mice

**Figure 8:**
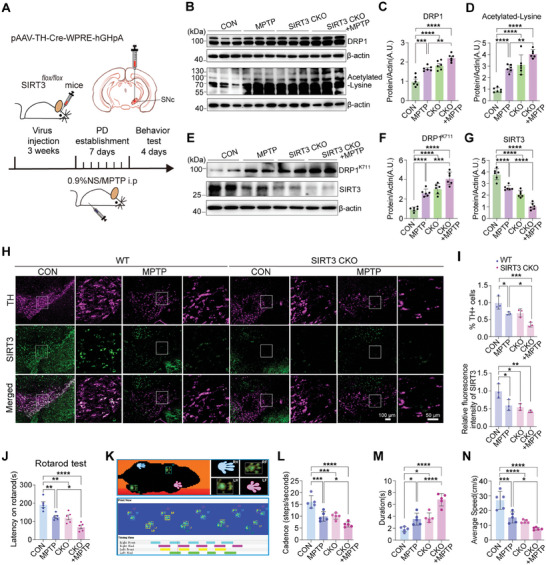
*SIRT3* knockout exacerbates dopaminergic neuron loss and motor deficits in a PD mouse model. A) Generation of TH‐specific *SIRT3*‐knocked‐out mice via stereotaxic injection of pAAV‐TH‐Cre‐WPRE‐hGHpA virus into the SNc of SIRT3^flox/flox^ mice. MPTP was administered to establish a PD mouse model, which was subjected to behavioral testing. B–D) Western blotting analysis of DRP1 and total acetylation levels in the SNc brain tissues of MPTP‐induced *SIRT3*‐knocked‐out PD mice. Data are represented as the mean ± SD (n = 6). **P < 0.01, ***P < 0.001 and ****P < 0.0001 versus indicated group. E–G) Western blotting analysis of SIRT3 and DRP1^K711^ levels in the SNc brain tissues of MPTP‐induced PD mice with *SIRT3* CKO. Data are represented as the mean ± SD (n = 6). ***P < 0.001 and ****P < 0.0001 versus indicated group. H,I) Immunofluorescence analysis of TH and SIRT3 expression levels in the SNc region of MPTP‐induced PD mice with *SIRT3* CKO. Data are represented as the mean ± SD (n = 3). *P < 0.05, **P < 0.01, and ***P < 0.001 versus indicated group. J) Rotarod test analysis of motor function in MPTP‐induced PD mice with *SIRT3* CKO. Data are represented as the mean ± SD (n = 5). *P < 0.05, **P < 0.01, and ****P < 0.0001 versus indicated group. K) Test diagram from the Catwalk system. The system shows green paw prints and records parameters after recognition. L–N) Catwalk gait analysis of cadence, duration, and average speed in MPTP‐induced PD mice with *SIRT3* CKO. Data are represented as the mean ± SD (n = 5). *P < 0.05, ***P < 0.001, and ****P < 0.0001 versus indicated group. Statistical analysis results are presented in Table S1 (Supporting Information).

Our data revealed that the activation or overexpression of SIRT3 protects against neuronal dysfunction in PD by regulating K711 acetylation of DRP1. To confirm the role of SIRT3 in regulating DRP1^K711^ acetylation, we generated a *SIRT3* conditional knockout (CKO) mouse by injecting pAAV‐TH‐Cre‐WPRE‐hGHpA virus into the SNc of SIRT3^flox/flox^ mice (verified via genotyping; Figure S12, Supporting Information) *SIRT3* CKO and control mice were further treated with MPTP to generate PD mouse models (**Figure** **8**A). *SIRT3* CKO mice exhibited impaired rotarod performance compared to the control animals (Figure S13, Supporting Information).

Western blotting analysis of SNc brain tissues of *SIRT3* CKO mice revealed MPTP‐induced decreased SIRT3 levels and increased total acetylated lysine and DRP1 levels, including acetylation at K711. These elevations were exacerbated in CKO mice due to increased mitochondrial fission (Figure 8B–G). Gliosis is also involved in the pathogenesis of PD. We did not observe significant changes in the astrocytes or microglia immunostained for GFAP and Iba1 in the SNc brain sections of *SIRT3* knockout mice (Figure S14, Supporting Information). Immunohistochemical staining of SNc brain tissues for SIRT3 and TH revealed that the knockout of *SIRT3* exacerbated the decrease in SIRT3 expression and loss of TH‐positive neurons in CKO mice (Figure 8H,I).

Rotarod testing revealed that the conditional knockout of *SIRT3* exacerbated the MPTP‐induced decrease in the latency to fall (Figure 8J). In the gait test, conditional *SIRT3* knockout exacerbated the MPTP‐induced decrease in cadence and average speed and increase in platform time (Figure 8K–N). These findings highlight the significant impact of *SIRT3* knockout on motor function in MPTP‐induced PD.

SIRT3 ablation exacerbates PD‐like pathology in mice, which is characterized by mitochondrial dysfunction, neuronal death, and behavioral deficits. Our findings suggest that SIRT3 protects against DA neuronal loss by preserving the mitochondrial morphology and functions. Mechanistically, SIRT3 deacetylation of DRP1 at K711 played key roles in these protective effects, suggesting its potential as a novel therapeutic target for PD.

## 3 Discussion

Mitochondrial dysfunction plays a crucial role in PD pathogenesis. DRP1, a key protein involved in mitochondrial fission, is associated with PD. DRP1 activity is tightly regulated by PTMs, such as phosphorylation and acetylation. This study revealed a novel regulatory mechanism for the acetylation of DRP1 at K711, providing insights into the pathogenesis and facilitating the targeted treatment of PD.

Mitochondria, often referred to as “cellular powerhouses”, are unique organelles with their unique genome. They are dynamic entities that undergo constant fusion and fission to adapt to energy demands and environmental stress. Fusion enables mitochondria to compensate for each other's defects by sharing their components. Conversely, fission not only regulates morphology and facilitates mitochondrial trafficking but also segregates the most severely damaged mitochondria, maintaining the health of the mitochondrial network. Under stress, if damage cannot be compensated for by fusion or eliminated by fission, it may lead to neuronal death and neurodegenerative disorders.^[^ ^44^ ^]^ Abnormalities in mitochondrial fusion and fission have been implicated in both familial and sporadic forms of PD.^[^ ^4^, ^45^, ^46^ ^]^ The machinery for mitochondrial fission and fusion is regulated by proteolysis and PTMs.^[^ ^47^ ^]^ Abnormal PTMs in aggregate‐prone proteins are among the main drivers of PD.^[^ ^48^ ^]^ Except highly involvement of protein phosphorylation in the pathogenesis of neurodegenerative diseases,^[^ ^27^, ^48^, ^49^, ^50^, ^51^ ^]^ acetylation modifications may also play a crucial role in their occurrence and progression. Notably, mitochondrial acetylation has been widely implicated in the regulation of mitochondrial protein homeostasis, including energy storage and utilization.^[^ ^20^ ^]^ Although PTMs and excessive activation of DRP1 are involved in PD‐related mitochondrial fission,^[^ ^28^, ^29^, ^52^, ^53^ ^]^ the effects of DRP1 acetylation on mitochondrial function and dynamics have not been extensively investigated.^[^ ^37^, ^54^, ^55^ ^]^ Our research revealed a critical acetylation site on K711 in DRP1 that independently regulated pathological mitochondrial fission under oxidative stress conditions. The DRP1^K711^ site resides within the GED) of DRP1, a region essential for stimulating DRP1 GTPase activity, facilitating high‐order complex formation, and ensuring efficient mitochondrial fission.

Given that protein acetylation is sensitive to the intracellular redox state, the acetylation of Drp1 may represent a pivotal event in Drp1 activation during oxidative stress, subsequently altering its function and leading to mitochondrial morphological and functional damage. We found that acetylation at DRP1^K711^ promoted DRP1 oligomerization and phosphorylation at S616, a modification known to enhance mitochondrial translocation and enzymatic activity,^[^ ^4^, ^49^, ^54^, ^56^ ^]^ leading to excessive DRP1 activation, mitochondrial fragmentation, functional impairment, and cellular apoptosis under oxidative conditions. Early studies linked the crosstalk between acetylation and phosphorylation, which can exhibit complex regulatory effects.^[^ ^57^ ^]^ Our study indicated that acetylation at K711 promotes phosphorylation at S616, increases DRP1 oligomerization, and produces effects consistent with the damage caused by increased S616 phosphorylation. Furthermore, mitochondrial damage caused by K711Q was not mitigated by the S616A mutation, and the S616D mutation did not affect the protective effect of K711R on mitochondrial morphology and function. Therefore, we propose that increased K711 acetylation synergizes with S616 phosphorylation to promote mitochondrial fission and exacerbate mitochondrial damage under oxidative stress. Additionally, K711 acetylation appears to play a unique regulatory role in mitochondrial dynamics, as its proliferation effects are independent of S616 phosphorylation. However, the complex mechanisms underlying the joint regulatory effect require further investigation.

A previous study demonstrated that the GED domain plays a crucial role in both the inter‐and intramolecular interactions of DRP1 GTPase, facilitating the formation and disassembly of complex structures.^[^ ^30^ ^]^ Our study confirmed that the acetylation site K711 of DRP1 is located within the GED domain, and its acetylation mutation promotes mitochondrial aggregation and oligomer formation. Therefore, we speculate that, under oxidative stress, increased K711 acetylation promotes the recruitment of cytoplasmic DRP1 to the mitochondria, further promoting its assembly into large oligomers, thereby initiating enhanced fission events that affect mitochondrial morphology and function, ultimately leading to cell damage. Interestingly, although it was not in a PD model, some studies have reported on the K642 site in a cardiac disease model.^[^ ^16^ ^]^ Both sites are located within the GED domain, suggesting that post‐translational modifications within the GED structure may affect the function of DRP1. However, the interaction between these sites still requires further exploration.

SIRT3, a member of the sirtuin family^[^ ^58^, ^59^, ^60^ ^]^ is involved in various aspects of mitochondrial metabolism and homeostasis and protects mitochondria from damage.^[^ ^61^ ^]^ It exerts neuroprotective effects in various PD models,^[^ ^21^, ^62^, ^63^, ^64^, ^65^ ^]^ although the underlying mechanisms are not fully understood. Our findings indicated that SIRT3 plays a crucial role in the regulation of mitochondrial acetylation. The genetic or pharmacological inhibition of SIRT3 leads to mitochondrial dysfunction. We found that SIRT3 directly interacted with DRP1 and specifically deacetylated the K711 site. SIRT3 overexpression protects against oxidative stress‐induced mitochondrial dysfunction by reducing DRP1^K711^ acetylation, thereby promoting mitochondrial health and reducing apoptosis. Our results indicate that SIRT3 reduces DRP1 acetylation at K711, decreasing DRP1 recruitment to the mitochondria and oligomer formation, thus improving mitochondrial morphology and function. This suggests that regulation of K711 acetylation can protect DA neurons from oxidative stress‐induced death. Consistent with our hypothesis, the SIRT3 agonist, HKL, reversed MPP+‐induced mitochondrial dysfunction, which was characterized by increased DRP1 acetylation at K711. In contrast, SIRT3 deficiency exacerbated MPP+‐induced mitochondrial dysfunction. These findings were further supported by in vivo studies using mouse models of PD. Treatment with the SIRT3 agonist HKL reversed MPTP‐ or 6‐OHDA‐induced increases in DRP1^K711^ acetylation, ameliorated behavioral impairments, and protected against DA neuron loss. Conversely, *SIRT3* CKO mice exhibit exacerbated mitochondrial damage, DA neuron loss, and behavioral impairments, confirming the detrimental effects of SIRT3 deficiency in vivo.

As a reversible protein deacetylase, SIRT3 regulates the PTMs of critical proteins involved in various mitochondrial processes. Normal mitochondrial fission/fusion maintains healthy mitochondrial networks. Abnormal fission caused by DRP1 induces excessive ROS production and triggers the caspase‐3‐dependent death pathway by releasing cytochrome c into the cytoplasm.^[^ ^66^ ^]^ In various disease models, inhibition of DRP1 activation reverses these changes and restores the mitochondrial function and morphology.^[^ ^67^, ^68^, ^69^ ^]^ This study confirmed the crucial role of SIRT3 in protecting DA neurons by regulating DRP1^K711^ acetylation in a PD model. In PD, decreased SIRT3 expression due to oxidative stress increased DRP1^K711^ acetylation, promoting DRP1 recruitment and oligomerization in the outer mitochondrial membrane and exacerbating mitochondrial fragmentation, leading to membrane potential depletion, impaired respiratory capacity, and increased ROS levels. Excessive ROS‐mediated modifications, such as DRP1 sulfonation at Cys644, further induce mitochondrial fragmentation and worsen the mitochondrial damage.^[^ ^70^ ^]^ In Parkinson's disease, the decline of SIRT3 may result from multiple factors. Alterations in the transcriptional regulation of the SIRT3 gene, such as changes in the activity of PGC – 1α, may lead to a reduction in the SIRT3 level. Additionally, it is possible that some ubiquitin‐ligases target SIRT3 to facilitate more efficient degradation. Further studies are necessary to determine whether other DRP1 modifications collective regulate its conformation and function at the K711 site, thereby affecting the mitochondrial dynamics.

**Figure 9:**
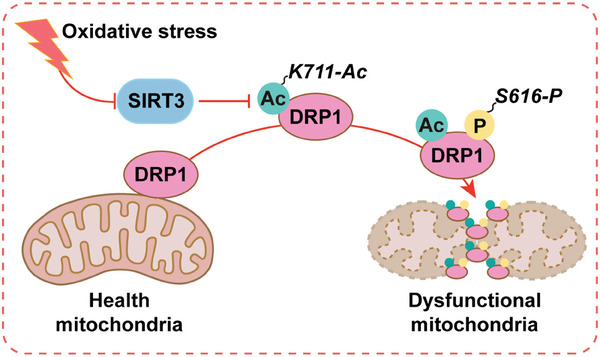
Schematic illustration of SIRT3‐mediated DRP1 acetylation is a cause of mitochondrial dysfunction and subsequent damage to dopaminergic neurons in Parkinson's disease.

Currently, the treatment of PD may encounter issues such as potential off‐target activities, restricted bioavailability, and difficulties in achieving the most appropriate dosing regimens. Meanwhile, the challenge of translating our research into clinical therapy is that it is difficult to precisely intervene the DRP1 acetylation site in the SNc region. Therefore, our study on the small molecule peptides and the upstream degradation pathways of SIRT3 could potentially provide alternative approaches. Collectively, we revealed that increased acetylation at the newly identified site K711 on DRP1 promotes mitochondrial fragmentation, leading to mitochondrial dysfunction and subsequent damage to dopaminergic neurons (**Figure** **9**). Our data suggest targeting the DRP1^K711^ acetylation site as a promising strategy for PD treatment. PD can also be treated by enhancing SIRT3 activity via pharmacological modulation or using therapeutic agents to directly inhibit DRP1 acetylation at K711. Further investigation of the roles of SIRT3 in other neurodegenerative diseases involving mitochondrial dysfunction will provide novel insights into the therapeutic potential of this target. This study highlights the intricate interplay among mitochondrial dynamics, SIRT3 activity, and PD pathogenesis, providing a promising new target for PD therapy.

## 4 Conclusion

In this study, we found that under oxidative stress, DRP1 acetylation at K711 in mitochondria increases. This promotes DRP1 oligomerization, leading to mitochondrial fragmentation and dysfunction, damaging DA neurons. We identified SIRT3 as a key regulator of DRP1K711 deacetylation. Pharmacological activation of SIRT3 can rescue DA neuronal loss and improve motor function in PD models. Conversely, SIRT3 knockout exacerbates DA neuronal damage. Targeting DRP1K711 acetylation or enhancing SIRT3 activity shows potential as a therapeutic strategy for PD.

## 5 Experimental Section

### Cell Culture and Treatment

HeLa human epithelial cervix carcinoma cells were cultured in Dulbecco's minimum essential medium (Gibco, USA) supplemented with 10% fetal bovine serum (FBS) (Gibco) at 37 °C with 5% CO_2_. SH‐SY5Y human neuroblastoma cells were cultured in a 1:1 mixture of Eagle's minimum essential medium (ATCC, USA) and Ham's F‐12 (F12; Gibco) supplemented with 10% FBS at 37 °C with 5% CO_2_. HeLa and 293T cells (obtained from Prof. Dayun feng, Fourth Military Medical University) were treated with H_2_O_2_ and MPP^+^ (MCE, USA) at the indicated time points and doses.

### Human iPSC Differentiation

Experiments used four human iPSC lines. Feeder‐free hiPSCs colonies were grown on matrigel and maintained in mTeSR plus (StemCell, Catalog No. 05825) with daily media changes. Floor plate (FP)‐cell‐based DA neuron induction was performed as previously described.^[^ ^71^, ^72^ ^]^ Briefly, hiPSC colonies were dissociated into single cells using accutase (StemCell, Catalog No. 07920) and seeded on matrigel‐coated plates with 1× ROCK inhibitor supplement (Selleckchem, Catalog No. S1049), then cultured with FP cell induction N1 medium supplemented with SB431542 (Tocris, Catalog No. 1614), 100 nm LDN‐193189 (Miltenyi Biotec, Catalog No. 130‐106‐540), 100 ng mL^−1^ SHH‐C24 (Peprotech, Catalog No. 100–45), 100 ng mL^−1^ FGF8 (MCE, Catalog No. HY‐P70533) and 2 µm Purmorphamine (MCE, Catalog No. HY‐15108). After five days, the culture medium was gradually switched to N2 medium supplemented with CHIR99021(Tocris, Catalog No. 4423) from day 6 to day 12 by mixing N1 and N2 in specific ratios on different days. FP cells were expanded in an N2 medium with 1× ROCK inhibitor supplement, frozen, and stored. After 6 passages of expansion, FP cells were dissociated into single cells with accutase, plated on freshly matrigel‐coated plates, and the medium was changed to DA induction medium containing Neurobasal medium/B27 (Gibco, Catalog No. 17504‐044) supplemented with GDNF, BDNF, ascorbic acid, DAPT, cAMP and transforming growth factor β3. Cells were then dissociated with accutase, diluted, and replated on dishes precoated with poly‐*D*‐lysine hydrobromide (Sigma, Catalog No. p7405) and laminin (Sigma, Catalog No. L2020) in DA differentiation medium until the desired maturation stage.

### Mito‐RFP, Plasmids, and siRNA Transfection

Mito‐RFP, plasmids, and siRNAs were transfected into cells using Lipofectamine 2000 (11668019; Invitrogen, USA), according to the manufacturer's instructions. The plasmid constructs were supplied by Tsingke Biotech Co., Ltd. (Beijing, China). cDNA of *Homo sapiens* DRP1 was synthesized based on its mRNA sequence (NM_001278464.2) and cloned into the pCMV‐C‐FLAG vector. Then, cDNAs encoding the DRP1 K711R, DRP1 K711Q, DRP1 K711R S616D, DRP1 S616D, DRP1 S616A and DRP1 K711Q S616A mutants were synthesized with the indicated nucleic acid adjustments (AAA711 replaced by AGA711, AAA711 replaced by CAA711, AGT616 replaced by GAT616, and AGT616 replaced by GCT616, respectively) and cloned into the pCMV‐C‐Flag vector. The cDNA of *H. sapiens* SIRT3 was synthesized based on its mRNA sequence (NM_012239.6). DRP1‐FLAG‐EYFP and SIRT3‐HA‐CFP were cloned into the pLVX‐puro vector for the FLIM‐FRET assay. DRP1 was cloned into the pBiFC‐VC155 vector and SIRT3 was cloned into pBiFC‐VN173 for BiFC assay. The following siRNAs were used: *siSIRT3* 5ʹ‐GGCUGCUUCUGCGGCUCUA‐3ʹ; *siSIRT5* 5ʹ‐ CCAGCUACGAACAGAUUCA‐3ʹ. Then, 293T cells expressing the control shRNA (pLKO.1‐puro) or shRNA targeting SIRT3 mRNA (HG13033‐CF; Sino Biological, China) were generated via lentivirus delivery and puromycin (5 µg mL^−1^) selection.

### Mitochondrial Isolation

Mitochondria were isolated from the cells using a mitochondrial isolation kit (C500051; Sangon Biotech, China), according to the manufacturer's protocol.

### Liquid Chromatography‐Tandem Mass Spectrometry (LC‐MS/MS) Analysis

Cell samples were collected and lysed using the radioimmunoprecipitation assay (RIPA) lysis buffer. Following lysis, the samples were centrifuged at 13 000 × *g* for 10 min, and the resulting supernatants were carefully collected to determine the protein concentration using the BCA assay. The BCA Protein Assay Kit (Bio‐Rad, Hercules, USA) was used for precise quantification. Subsequently, 40 µg of protein from each sample was mixed with the 5× loading buffer and boiled for 5 min. Proteins were separated on a polyacrylamide gel via 6–10% sodium dodecyl sulfate‐polyacrylamide gel electrophoresis. The gel was stained with Coomassie Blue G‐250 (Solarbio Life Sciences, China). The prepared samples were sent to Shanghai Applied Protein Technology, China for LC‐MS analysis. The Mascot 2.2 software was used to match the experimental mass values to specific peptide sequences within the UniProt database.

### MitoTracker Labeling and Mitochondrial Membrane Potential Measurement

HeLa or SH‐SY5Y cells were stained with 10 nm MitoTracker Red CMXRos (Life Technologies, USA) at 37 °C for 30 min. Mitochondria were observed using the Olympus FV3000 confocal microscope. Mitochondrial length analysis was performed using a sample of 50 randomly selected cells within each group with the ImageJ software. The analysis followed the aspect ratio and form factor methods as previously described.^[^ ^73^ ^]^ The cells were treated with 10 nm TMRE (Life Technologies) in Hank's balanced salt solution (Life Technologies) at 37 °C for 30 min. After incubation, the cells were washed thrice with phosphate‐buffered saline to remove excess TMRM, according to the manufacturer's instructions. Pharmacological controls included cells exposed to 10 µm carbonyl cyanide p‐trifluoromethoxyphenylhydrazone (FCCP; Sigma, USA) in Hank's balanced salt solution at 37 °c for 30 min. The stained samples were examined using a fluorescence microscope (FV3000; Olympus, Japan) with TRITC/REF filter settings.

### ROS Detection

ROS levels were evaluated using 2′,7′‐dichlorofluorescein‐diacetate with the ROS Assay Kit (Nanjing Jiancheng Bioengineering Institute, China). The cells were treated with 10 µm 2′,7′‐dichlorofluorescein‐diacetate at 37 °C for 30 min and observed via confocal fluorescence microscopy, according to the manufacturer's guidelines.

### Western Blotting

Cells and tissue samples of the substantia nigra were lysed using the RIPA lysis buffer (Beyotime, China) supplemented with protease and phosphatase inhibitors (43002700; 41659200; Roche, Switzerland), incubated for 30 min on ice, and centrifuged at 12 000 × *g* at 4 °C. The supernatant was collected and mixed with 5× loading buffer, followed by incubation at 95 °C for 5 min in a metal bath to prepare the protein sample. Equivalent protein amounts were subjected to sodium dodecyl sulfate‐polyacrylamide gel electrophoresis, transferred to polyvinylidene difluoride membranes (Merck Millipore, Germany), blocked with 5% bovine serum albumin, and incubated with specific primary antibodies at 4 °C overnight. After incubating again with the corresponding secondary antibodies for 2 h, the reaction bands were visualized using an enhanced chemiluminescence reagent (Merck Millipore). Subsequently, semi‐quantification of protein band density was performed using the ImageJ software. The following primary antibodies were used: anti‐SirTt3 (1:1000; D22A3; Cell Signaling Technology, USA), anti‐DRP1 (1:1000; ab184247; Abcam, England), anti‐phospho‐DRP1 (Ser616) (1:1000; 3455S; Cell Signaling Technology), anti‐phospho‐(Ser/Thr) Phe (1:1000; 9631S; Cell Signaling Technology), anti‐voltage‐dependent anion channel (1:1000; 4661S; Cell Signaling Technology), anti‐Flag (1:1000; 66008‐3‐Ig; 20543‐1‐AP; Proteintech, USA), anti‐acetyl lysine (1:1000, ab22550, ab190479; Abcam), anti‐β‐actin (1:50 000; AC026; ABclonal, USA), and anti‐DRP1^K711^(1:1000; PTM BioLab [Hangzhou] Co. Ltd, China) antibodies.

### HIS‐Pulldown Assay for Recombinant Protein Interaction

The recombinant protein DRP1 purified by Nanjing Detai Biotechnology Co., Ltd. was used. For its expression and purification, pET30a (with a target sequence of N – HIS) was chosen as the vector and E. coli as the host cell. The recombinant Sirt3 protein, sourced from MedChemExpress (HY‐P701609), was tag‐free. Two proteins were dissolved in IP lysis buffer (Thermo Fisher Scientific, USA) supplemented with protease and phosphatase inhibitors at 4 °C for interaction over 5 h. Then, part of the mixture was used to prepare the input sample following the Western blotting sample preparation protocol. The remaining portion was combined with HIS‐tagged or non‐HIS‐tagged magnetic beads and incubated for 4 h at 4 °C prior to sample preparation. Subsequently, the samples were subjected to electrophoresis on an SDS – PAGE gel. After electrophoresis, the gel was stained with Coomassie Brilliant Blue for protein visualization.

### Co‐IP Assay

Utilizing a blender, cell samples were lysed at 4 °C for 4 h in IP lysis buffer (Thermo Fisher Scientific, USA) supplemented with protease and phosphatase inhibitors. After centrifugation at 4 °C and 13 000 rpm for 10 min, the supernatant was incubated overnight at 4 °C with primary antibodies or non‐specific IgG. After the introduction of protein A/G magnetic beads into the protein lysate, the mixture was agitated on a rotator at 4 °C for 4 h. The beads subsequently isolated using a magnetic rack, washed thrice with the Co‐IP lysis buffer, resuspended in loading buffer, and used for subsequent experiments.

### DRP1 Oligomerization Assay

After the designated treatments, cell pellets were taken into 1.5 mL microcentrifuge tubes and agitated in the presence of disuccinimidyl suberate (A39267; Thermo Fisher Scientific) at a final concentration of 4 mm at room temperature for 30 min. Subsequently, Tris‐HCl (pH 7, 20 mm) was introduced into the tubes, followed by 15‐min incubation in the dark. The samples were centrifuged at 1000 × *g* and 4 °C for 5 min to pelletize the crosslinked materials. The pellets were resuspended in RIPA buffer supplemented with protease and phosphatase inhibitors. Finally, western blotting was performed as previously described.

### FLIM‐FRET

In the FRET assay, cells were seeded in a confocal dish and transfected with DRP1‐EYFP or SIRT3‐CFP, as indicated. CFP was excited at 405 nm, with emission monitored in the range of 460–492 nm. In contrast, EYFP was excited at 514 nm, with emission in the range of 526–589 nm. For FLIM data analysis, SymPhoTime 64 (v2.6; PicoQuant GmbH, Germany), specialized software offering a dedicated FLIM‐based lifetime FRET mode was used for further investigation. The integrated microscope system used a high‐power 440 nm picosecond pulsed laser (LDH‐P‐C‐440B; PicoQuant GmbH), along with a tunable laser driver (PDL‐800‐D; PicoQuant GmbH) capable of adjusting pulse rates from 31.25 kHz to 40 MHz. The detection system was hybrid in nature and combined a photomultiplier tube and single‐photon avalanche diode qualities (PMA hybrid 40; PicoQuant GmbH). It exhibited a jitter of 120 ps and an efficiency of 45% at 500 nm. The core apparatus for time‐correlated single‐photon counting was a Picoharp 300 (PicoQuant GmbH), with an ultrafine timing resolution of 4 ps and high data throughput. This enabled the generation of fluorescence decay histograms based on the photon emission times relative to the laser excitation pulse, with interpulse intervals distributed across the image pixels.

### BIFC Assay

For BiFC analysis, the recombinant plasmids, pBiFC‐VC155‐DRP1 and pBiFC‐VN173‐ SIRT3, which were concomitantly transfected into 293T cells were used. These cells were seeded in a confocal dish to facilitate the assembly of the BiFC complexes. As negative controls, two groups were included: one transfected with pBiFC‐VC155 and pBiFC‐VN173‐SIRT3 plasmids, and the other co‐transfected with pBiFC‐VC155‐DRP1 and pBiFC‐VN173 plasmids. The transfected 293T cells were cultured in the Dulbecco's modified Eagle's medium supplemented with 5% FBS for 24 h at 37 °C. After 24‐h incubation, the cells were harvested and subjected to a 10‐min incubation with 4′,6‐diamidino‐2‐phenylindole dihydrochloride (DAPI) at 37 °C in the dark. Subsequently, the specimens were meticulously examined using the discerning lens of a fluorescence microscope.

### TUNEL Staining

TUNEL assay was performed using the In Situ Cell Death Detection Kit (Roche, Switzerland). Post‐treatment, the cells were fixed by 4% paraformaldehyde in 0.2 m phosphate buffer (pH 7.4) and incubated at 37 °C for 1 h with the TUNEL reaction mixture, according to the manufacturer's protocol. Nuclei were counterstained with DAPI and visualized using laser scanning confocal microscopy. The apoptotic rate was determined as the percentage of TUNEL‐positive cells in each group.

### Immunofluorescence Assay

Cell or tissue specimens were fixed with 4% paraformaldehyde for 20 min, permeabilized using 0.3% Triton X‐100, blocked with 5% bovine serum albumin, and incubated overnight at 4 °C with the appropriate primary antibodies. After washing with phosphate‐buffered saline, the sections underwent 2 h incubation at room temperature with the corresponding secondary antibodies and DAPI reagent, followed by visualization using a fluorescence microscope. The following primary antibodies were used: anti‐TOM20 (1:100; ab186734; Abcam), anti‐TH (1:5000; HPA061003; Sigma‐Aldrich, USA); anti‐SirT3 (1:300; D22A3; Cell Signaling Technology), anti‐DRP1 (1:300; ab184247; Abcam), and anti‐acetyl lysine (1:200; ab22550; ab190479; Abcam) antibodies.

### Measurement of OCR

OCR assays were performed using the Seahorse XF‐24 extracellular flux analyzer (Seahorse Bioscience). One day prior to the measurements, the cells were seeded in a 24‐well XF microplate (Agilent Technologies Inc., USA) at a density of 8 × 10^6^ cells well^−1^ and subsequently treated as per the respective experimental protocols. During the experiments, specific compounds, oligomycin (1 µm), FCCP (1 µm), and rotenone (1 µm), derived from the Seahorse XF Cell Mito‐stress Test Kit (103015–100; Agilent Technologies Inc) were administered at specific time points. Post‐experiment data analysis was conducted using the Wave Desktop Software (Agilent Technologies Inc.).

### Transmission Electron Microscopy

Mice underwent intracardial perfusion with a fixative solution composed of 2% paraformaldehyde and 2% glutaraldehyde for 1 h. Following perfusion, brain tissues were immersed in 2.5% glutaraldehyde at room temperature for 2 h, washed thrice in 0.1 m phosphate buffer (pH 7.4, prepared with KH_2_PO_4_ and Na_2_HPO_4_ from Sigma‐Aldrich), and fixed with 1% osmium tetroxide for an additional 2 h at room temperature, followed by washing with phosphate buffer. Subsequently, the samples were treated with a 0.1 m Pb solution, subjected to graded alcohol dehydration (30–100%), and embedded in a 1:1 mixture of acetone and Epon812 for 45 min, followed by polymerization at 60 °C for 24 h. Serial 70 nm ultrathin sections from the SNc regions were stained with a solution containing 3% uranyl acetate and lead citrate. The mitochondrial morphology of SNc neurons was examined using the JEM‐1230 transmission electron microscope (Japan Electron Optics Laboratory, Tokyo, Japan). Images were captured using a CCD camera (Olympus). Subsequent analysis of mitochondrial morphology parameters was performed using the ImageJ software.

### Animals

TH‐Cre mice (B6. [Cg‐7630403G23RikTg (Th‐cre)1Tmd/J]), SIRT3^flox/flox ^(B6.129 [Cg‐SIRT3tm1.1 Auw/J]) mice were purchased from the Jackson Laboratory, USA. C57BL/6J mice were obtained from the Experimental Animal Center of the Fourth Military Medical University, Xi'an, China. All experimental protocols were approved by the Committee of Animal Care and Use of the Fourth Military Medical University and adhered to the Animal Research: Reporting of In Vivo Experiments guidelines (FMMULL‐20220930). In an environment of constant temperature, the mice were kept under a 12‐h light/dark cycle, provided ad libitum access to food and water, group‐housed with 2–5 littermates until surgery, and randomly assigned into groups. Transgenic mice were distinguished via polymerase chain reaction (PCR) analysis of genomic DNA extracted from the tail samples using the following primer sequences: *SIRT3^flox^* ^/flox^ forward primer (5′‐CTGGCTTTGGGTTTAAGCAG‐3′) and reverse primer (5′‐GGAGGCTGAGGCTAAAGAGC‐3′). Subsequently, PCR products were assessed via electrophoresis on a 1.2% agarose gel.

### Viruses

pAAV‐hSyn‐mCherry‐3FLAG‐WPRE (serotype 2/9, titer 2 × 10^13^ vector genome per mL [vg mL^−1^]), pAAV‐TH‐Cre‐WPRE‐hGHpA (serotype 2/9, titer 2 × 10^13^ vector genome per mL [vg mL^−1^]), pAAV‐CMV‐DIO‐EGFP‐tWPA (serotype 2/9, titer 2 × 10^13^ vector genome per mL [vg mL^−1^]) and pAAV‐CMV‐DIO‐EGFP‐P2A‐DRP1(K711Q)‐3 × FLAG‐tWPA (serotype 2/9, titer 2 × 10^13^ vector genome per mL [vg mL^−1^]) were purchased from Obio Technology (Shanghai) Corp. Ltd., China.

### Stereotaxic Viral Injection

The mice were intraperitoneally anesthetized with 1% pentasorbital sodium for stereotaxic injection. Skull measurements were referenced to Bregma, and viruses were injected into the SNc (AP: −3.01 mm; ML: 1.2 mm; DV: −4.5 mm) using a 10‐µL microsyringe (Gaoge) delivering the virus at 30 nL min^−1^ via a microsyringe pump (Kd Scientific). For the CKO and control experiments, 300 nL of pAAV‐TH‐Cre‐WPRE‐hGHpA or pAAV‐hSyn‐mCherry‐3FLAG‐WPRE was bilaterally injected into the SNc. Moreover, 1 µL 6‐OHDA (1 µg µL^−1^) was injected into the SNc to establish the 6‐OHDA‐induced PD model. For the DRP1^K711^ overexpression mice and control experiments, 300 nL of pAAV‐CMV‐DIO‐EGFP‐tWPA or pAAV‐CMV‐DIO‐EGFP‐P2A‐DRP1(K711Q)‐3× FLAG‐tWPA was bilaterally injected into the SNc.

### Animal Models

For the establishment of the DRP1^K711Q^ overexpression mice model, the TH‐cre mice (8—10 weeks old) were randomly divided into the control group and the model group. Three weeks after the injection of the virus, subsequent experiments were carried out. MPTP‐induced male C57BL/6 mice (8–10 weeks old) with PD were randomly assigned to four groups: control, MPTP, *SIRT3* CKO, and *SIRT3* CKO + MPTP groups. The PD model was established via daily intraperitoneal injections of 30 mg kg^−1^ free‐base MPTP for seven consecutive days. The control and *SIRT3* CKO groups were administered equivalent volumes of 0.9% saline. The successful establishment of the PD model was confirmed via behavioral assessment and histological analysis of the substantia nigra. Then, *SIRT3* CKO mice were generated using the Cre‐lox system targeting the SNc. The efficiency of the knockout was confirmed via PCR and western blotting. HKL‐treated MPTP‐induced male C57BL/6 mice (8–10 weeks old) with PD were randomly assigned to four groups: control, MPTP, HKL, and HKL + MPTP groups. HKL (40 mg kg^−1^) was intraperitoneally injected 36 h before MPTP administration. MPTP (30 mg kg^−1^ free base) was injected daily for seven consecutive days. HKL‐treated 6‐OHDA‐induced male C57BL/6 mice (8–10 weeks old) with PD were randomly assigned to four groups: control, 6‐OHDA, HKL, and HKL + 6‐OHDA groups. HKL (40 mg kg^−1^) was intraperitoneally injected 36 h before 6‐OHDA administration for seven consecutive days.

### Gait Analysis

PD model rats were subjected to gait assessment using the CatWalk automated gait analysis system (Noldus Information Technology, Wageningen, Netherlands). This system consisted of an extensive glass platform illuminated by a fluorescent light source that precisely captures the footprints of rodents as they traverse a path. Prior to PD model induction, the mice underwent five days of unforced training, walking across the glass walkway at least thrice daily. Inadequate performance during the CatWalk training phase resulted in exclusion from the study. Subsequently, a three‐day unforced test was conducted, with the mice traversing the glass walkway at least thrice daily. Three consecutive, uninterrupted, and straight runs were selected per mouse for statistical analyses. Gait parameters, including cadence (number of steps per second), duration (total run time in seconds), and average speed (speed of the recorded run), were assessed using the CatWalk system (Noldus, Netherlands).

### Rotarod Test

Mice were subjected to a rotarod test using a programmable acceleration paradigm, which gradually increased the rotation speed from 4 to 40 rpm over a 90‐s interval. The primary objective was to measure the duration for which the mice remained on the rotating rod, with a maximum observation time of 300 s.The mice underwent a three‐day training phase before the test. On the initial day of training, the mice were introduced to the rotating rod set at a constant speed of 5 rpm, and any falls resulted in immediate reinstatement of the mice on the rod, which continued until 300 s. On the last training day, the mice were kept on the rotating rod, and the speed was progressively increased from 4 to 20 rpm. Each mouse participated in three rotarod sessions. The fourth day was marked as the official test day, as described above.

### Statistical Analyses

Statistical analyses were conducted using GraphPad Prism version 9.0 (GraphPad Software Inc., USA). Data normality was assessed using the Shapiro–Wilk test to ensure the validity of parametric tests. A two‐tailed unpaired *t*‐test was used to compare two experimental groups, whereas a one‐way analysis of variance followed by Tukey's multiple comparison test was used to compare multiple experimental groups. To investigate the effects of the two independent variables, a two‐way repeated‐measures analysis of variance was conducted. All experimental data were represented as the mean ± standard deviation. Each experiment was performed in triplicate (*n* ≥ 3). The specific numbers of replicates and statistical methods used for each analysis were described in the respective figure legends. Statistical analysis results are presented in Table S1 (Supporting Information). Statistical significance was set at *P* < 0.05.

## Conflict of Interest

The authors declare no conflict of interest.

## Supporting information



Supporting Information

Supplemental Table 1

## Data Availability

The data that support the findings of this study are available from the corresponding author upon reasonable request.
