# Supplementary material for: SIRT3‐Mediated Deacetylation of DRP1K711 Prevents Mitochondrial Dysfunction in Parkinson's Disease
Source: Adv Sci (Weinh). 2025 Feb 20;12(17):2411235. doi: 10.1002/advs.202411235 (PMC12061286; doi:10.1002/advs.202411235)
Supplement: Supplementary file 1 — Supporting Information [file ADVS-12-2411235-s002.docx]

**Supplementary Materia**l


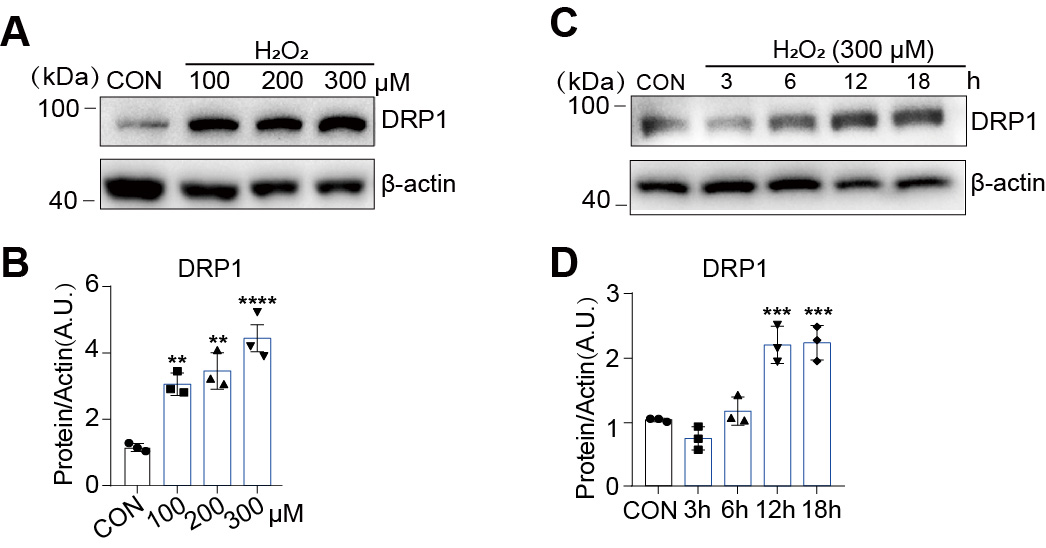


**Figure S1. Optimization of H_2_O_2_ treatment conditions for SH-SY5Y cells.** **(A–B)** Western blotting analysis of DRP1 expression in cells after dose-dependent treatment with H_2_O_2_. Data are represented as the mean ± SD (n = 3). ***P* < 0.01 and *****P* < 0.0001 *vs.* indicated group. **(C–D)** Western blotting analysis of DRP1 expression in cells after time-dependent treatment with H_2_O_2_. Data are represented as the mean ± SD (n = 3). ****P* < 0.001 *vs.* indicated group. Statistical analysis results are presented in Table S1.


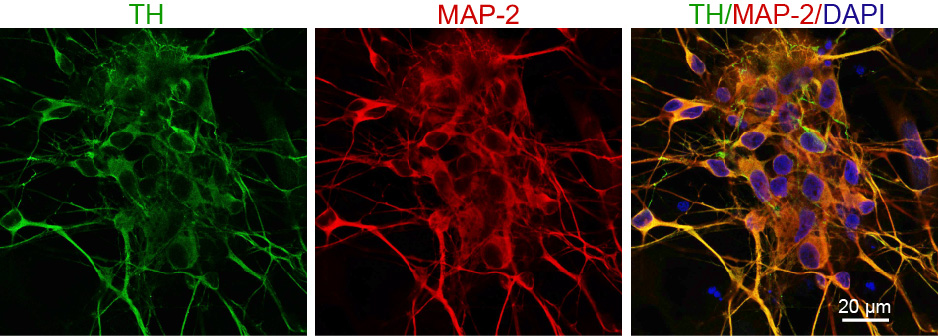


**Figure S2.** **Dopaminergic neurons derived from human induced pluripotent stem cell (hiPSCs).** Immunofluorescence images of TH and microtubule-associated protein 2 (MAP - 2) in DA neurons differentiated from human induced pluripotent stem cells (hiPSCs) on day 14. Scale bar: 20 μm.


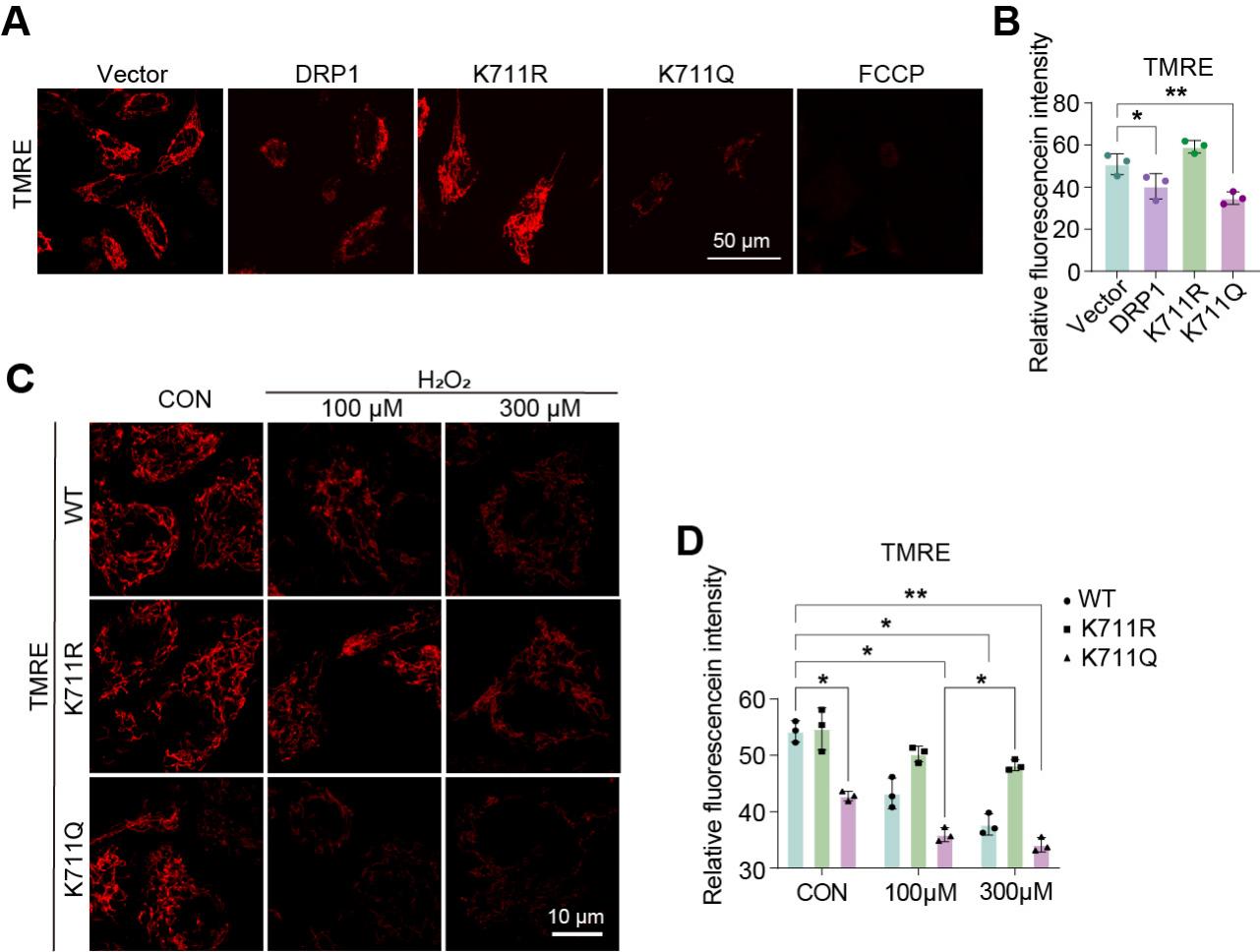


**Figure S3. Increased acetylation of DRP1^K711^ induced by oxidative stress reduces the mitochondrial membrane potential. (A–B)** TMRE staining and analysis of mitochondrial membrane potential in the control and DRP1, K711R, and K711Q mutant plasmid-transfected HeLa cells. Scale bar: 50 μm. Data are represented as the mean ± SD (n = 3). **P* < 0.05 and ***P* < 0.01 *vs.* indicated group. **(C–D)** HeLa cells transfected with the K711R and K711Q mutant plasmids were treated with 100 or 300 μM H_2_O_2_. (C) Representative images of TMRE staining. (D) Quantification of the relative fluorescence intensity of TMRE. Scale bar: 10 µm. Data are represented as the mean ± SD (n = 3). **P* < 0.05 and ***P* < 0.01 *vs.* indicated group. Statistical analysis results are presented in Table S1.


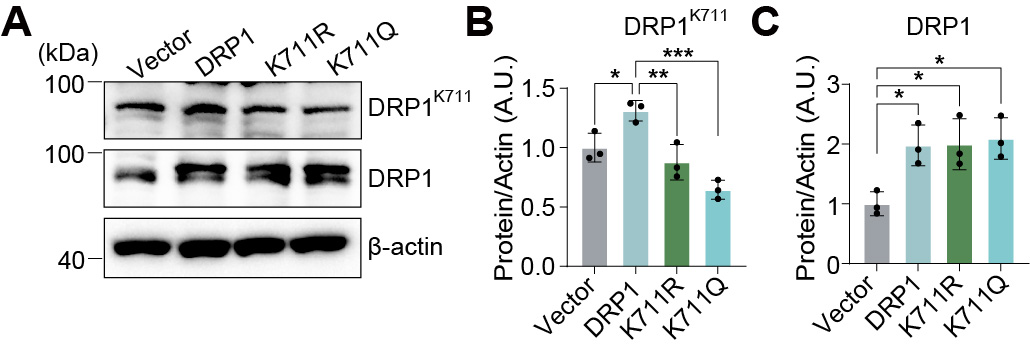


**Figure S4. Mutation at the K711 site can lead to a decrease in the expression level of DRP1^K711^. (A–C)** Western blotting analysis of DRP1 and DRP1^S616^ levels in HeLa cells transfected with plasmids encoding wild-type DRP1, K711R and K711Q. Data are represented as the mean ± SD (n = 3). **P* < 0.05, ***P* < 0.01 and ****P* < 0.001 *vs.* indicated group.


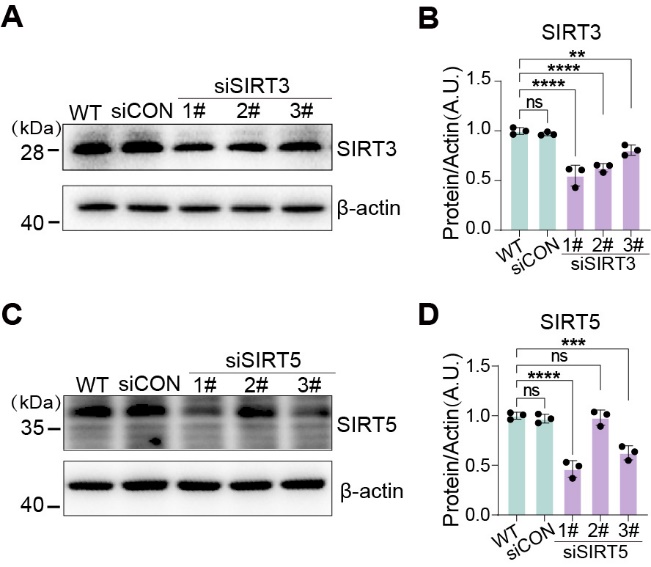


**Figure S5. Validation of siRNA knockdown efficiency. (A-B)** Western blotting analysis of the knockdown efficiencies of different siRNA sequences targeting SIRT3 in HeLa cells. Data are represented as the mean ± SD (n = 3). ***P* < 0.01 and *****P* < 0.0001 *vs.* indicated group. **(C–D)** Western blotting analysis of the siRNA knockdown specificity for SIRT5 by determining the SIRT5 protein levels. Data are represented as the mean ± SD (n = 3). ****P* < 0.001 and *****P* < 0.0001 *vs.* indicated group. *ns*, not signiﬁcant. Statistical analysis results are presented in Table S1.

**
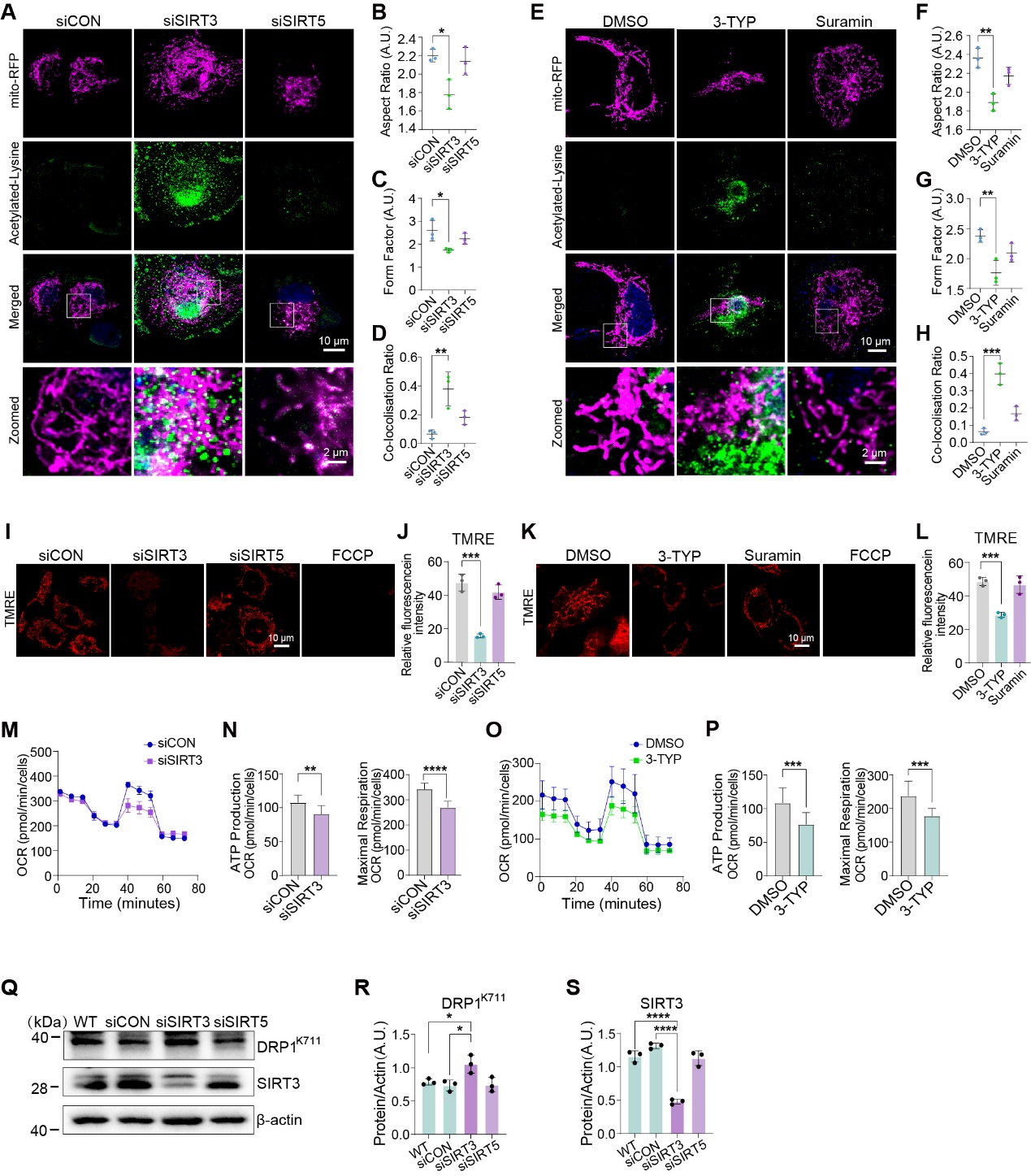
**

**Figure S6.** **SIRT3 is a crucial enzyme regulating DRP1 acetylation to influence mitochondrial morphology and functions. (A–D)** Immunofluorescence staining of HeLa cells treated with siRNAs targeting SIRT3 (siSIRT3) and SIRT5 (siSIRT5) and analysis of the effects on mitochondrial morphology, acetylation levels, and co-localization. Scale bars: 10 μm (main image); 2 μm (zoomed). Data are represented as the mean ± SD (n = 3). **P* < 0.05 and ***P* < 0.01 *vs.* indicated group. **(E–H)** Immunofluorescence staining of HeLa cells treated with the SIRT3 inhibitor, 3-TYP, and SIRT5 inhibitor, suramin, and analysis of the effects on mitochondrial morphology, acetylation levels, and co-localization. Scale bars: 10 μm (main image); 2 μm (zoomed). Data are represented as the mean ± SD (n = 3). ***P* < 0.01 and ****P* < 0.001 *vs.* indicated group. **(I–J)** Assessment of mitochondrial membrane potential in siRNA-transfected HeLa cells via TMRE staining. Scale bar: 10 μm. Data are represented as the mean ± SD (n = 3). ****P* < 0.001 *vs.* indicated group. **(K–L)** Assessment of mitochondrial membrane potential in HeLa cells treated with the SIRT3 and SIRT5 inhibitors via TMRE staining. Scale bar: 10 μm. Data are represented as the mean ± SD (n = 3). ****P* < 0.001 *vs.* indicated group. **(M–N)** Oxygen consumption rate (OCR) analysis of ATP levels and maximal respiratory capacity in HeLa cells transfected with siSIRT3. Data are represented as the mean ± SD (n = 4). ***P* < 0.01 and *****P* < 0.0001 *vs.* indicated group. **(O–P)** OCR analysis of ATP levels and maximal respiratory capacity in HeLa cells treated with 3-TYP. Data are represented as the mean ± SD (n = 4). ****P* < 0.001 *vs.* indicated group. **(Q–S)** Western blotting analysis of DRP1^K711^ levels in siRNA-transfected HeLa cells. Data are represented as the mean ± SD (n = 3). **P* < 0.05 and *****P* < 0.0001 *vs.* indicated group. Statistical analysis results are presented in Table S1.


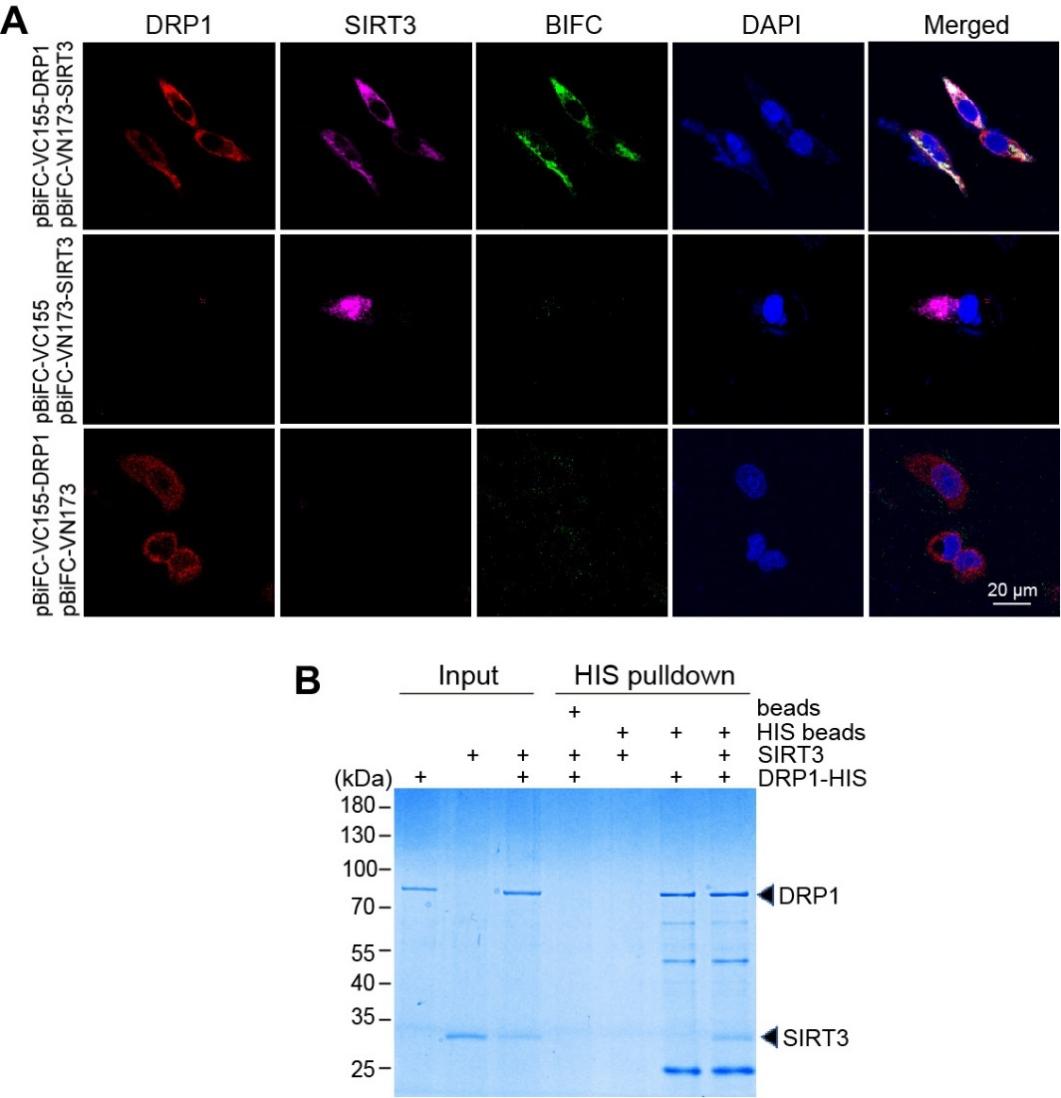


**Figure S7. Bimolecular fluorescence complementation (BiFC) assay and the recombinant protein HIS pulldown experiment confirmed the interaction between SIRT3 and DRP1. (A)** BiFC assay demonstrating the interaction between DRP1 and SIRT3 in HeLa cells. Magenta fluorescence indicates successful SIRT3 transfection, red fluorescence indicates successful DRP1 transfection, and green fluorescence indicates protein interaction. Scale bar: 20 µm. **(B)** Coomassie Brilliant Blue staining of DRP1-SIRT3 interaction (Recombinant Proteins, SDS-PAGE).


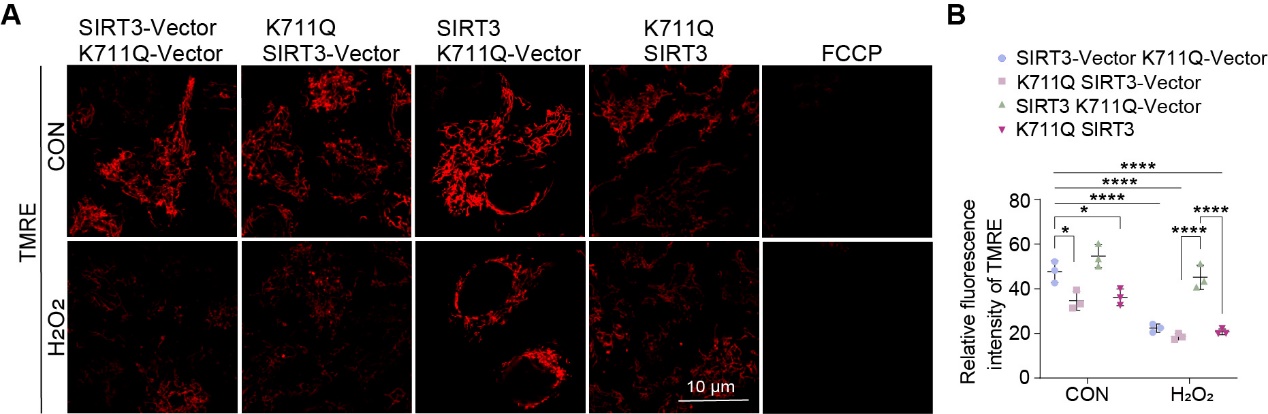


**Figure S8. Mitochondrial membrane potential in HeLa cells transfected with the SIRT3 and K711Q plasmids and subsequently treated with H_2_O_2_.**

**(A)** Representative TMRE staining images of HeLa cells transfected with the SIRT3 and K711Q plasmids and treated with H_2_O_2_ to assess mitochondrial membrane potential. Scale bar: 10 μm. **(B)** Quantification of the relative fluorescence intensity of TMRE for HeLa cells transfected with the SIRT3 and K711Q plasmids and treated with H_2_O_2_. Data are represented as the mean ± SD (n = 3). **P* < 0.05 and *****P* < 0.0001 *vs.* indicated group. Statistical analysis results are presented in Table S1.


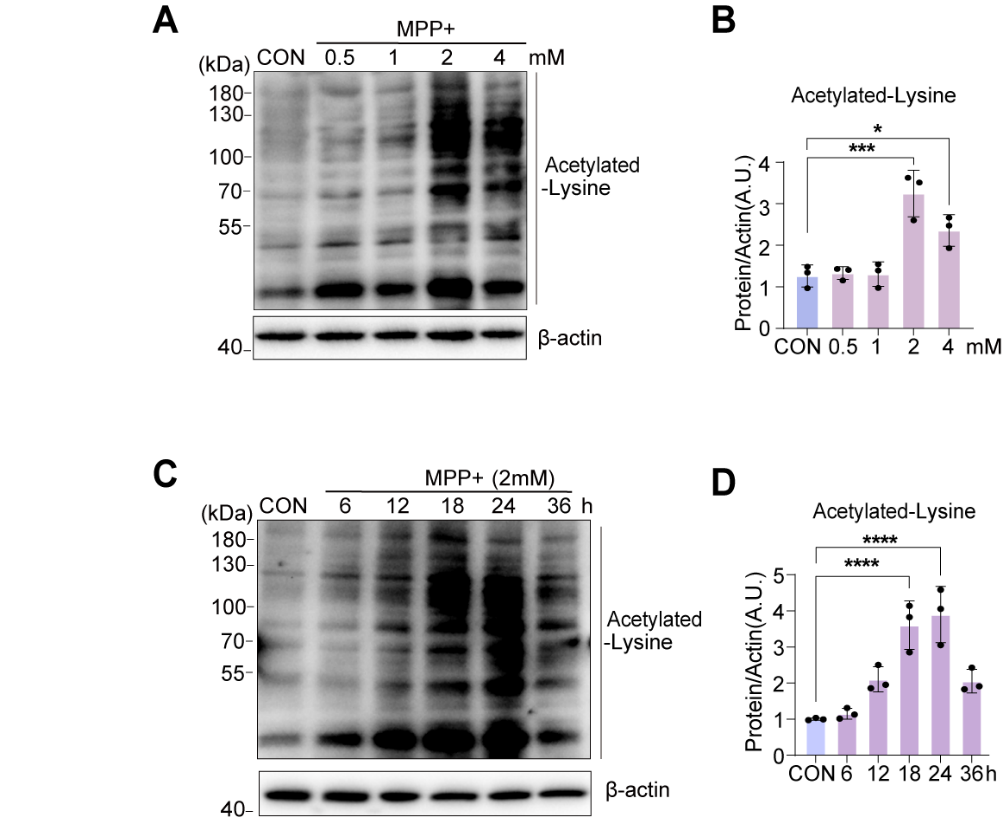


**Figure S9.** **Optimization of MPP^+^ treatment dosage and duration for SH-SY5Y cells. (A–B)** Western blotting analysis of acetylated-lysine expression in SH-SY5Y cells treated with different doses of MPP^+^ (0, 0.5, 1 and 2 mM) for 24 h. Data are represented as the mean ± SD (n = 3). **P* < 0.05 and ****P* < 0.001 *vs.* indicated group. **(C–D)** Western blotting analysis of acetylated-lysine expression in SH-SY5Y cells treated with 2 mM MPP^+^ for different durations (0, 6, 12, 18, 24, and 36 h). Data are represented as the mean ± SD (n = 3). *****P* < 0.0001 *vs.* indicated group. Statistical analysis results are presented in Table S1.

**
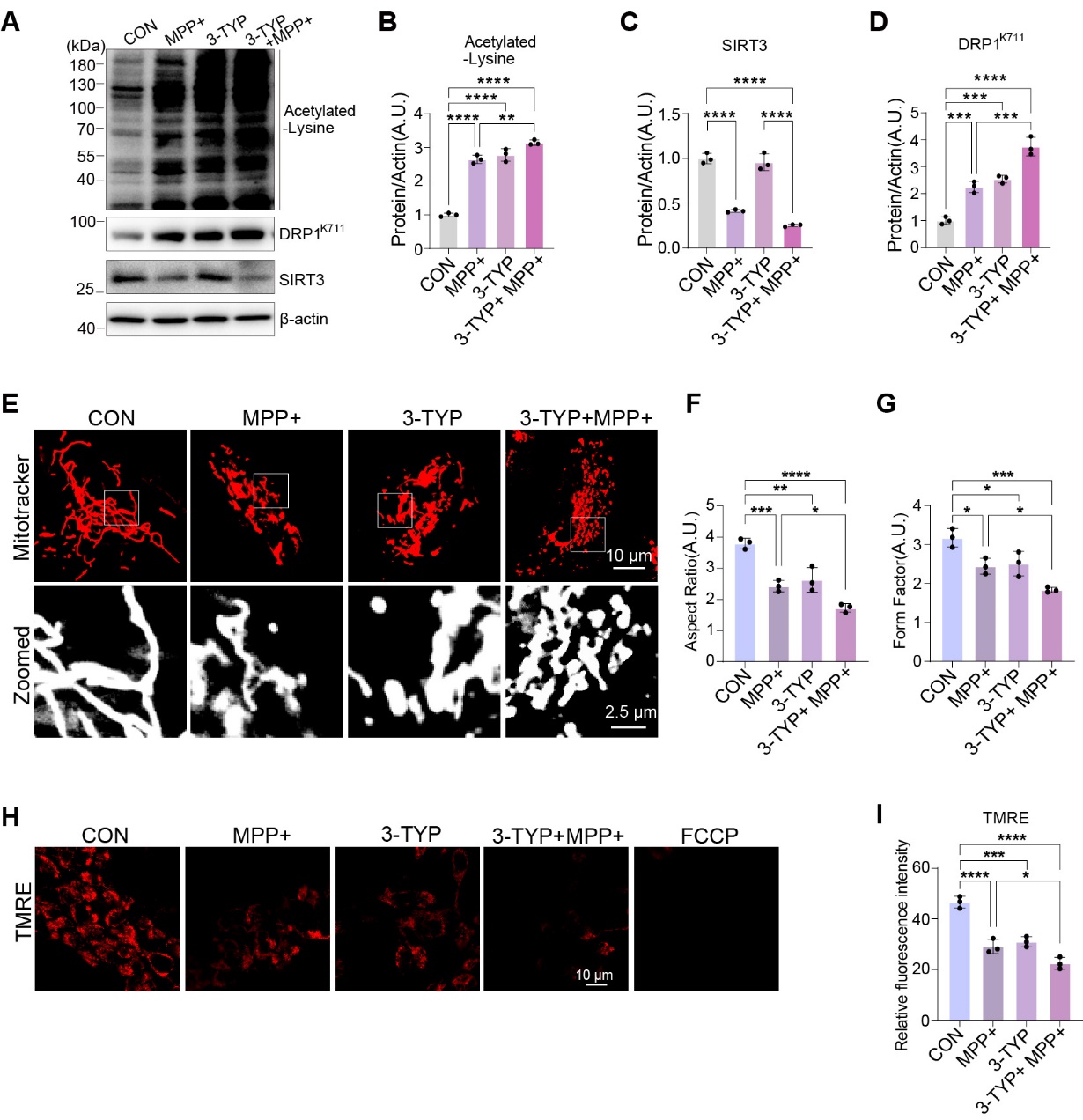
**

**Figure S10. SIRT3 inhibition aggravates the mitochondrial morphology and function damage in a PD cell model. (A–D)** Western blotting analysis of the effects of SIRT3 inhibitor 3-TYP on the acetylated lysine, DRP1^K711^, and SIRT3 levels in SH-SY5Y cells after MPP^+^ treatment. Data are represented as the mean ± SD (n = 3). ***P* < 0.01, ****P* < 0.001, and *****P* < 0.0001. **(E–G)** MitoTracker staining to assess mitochondrial morphology in vivo in the 3-TYP-treated PD cell model. Aspect ratio and form factor of mitochondria were quantified. Scale bar: 10 μm (main image); 2.5 μm (zoomed). Data are represented as the mean ± SD (n = 3). **P* < 0.05, ***P* < 0.01, ****P* < 0.001, and *****P* < 0.0001 *vs.* indicated group. **(H–I)** Mitochondrial membrane potential in SH-SY5Y cells assessed by measuring the fluorescence intensity of TMRE. Scale bar: 10 μm. Data are represented as the mean ± SD (n = 3). **P* < 0.05, ****P* < 0.001, and *****P* < 0.0001 *vs.* indicated group. Statistical analysis results are presented in Table S1.


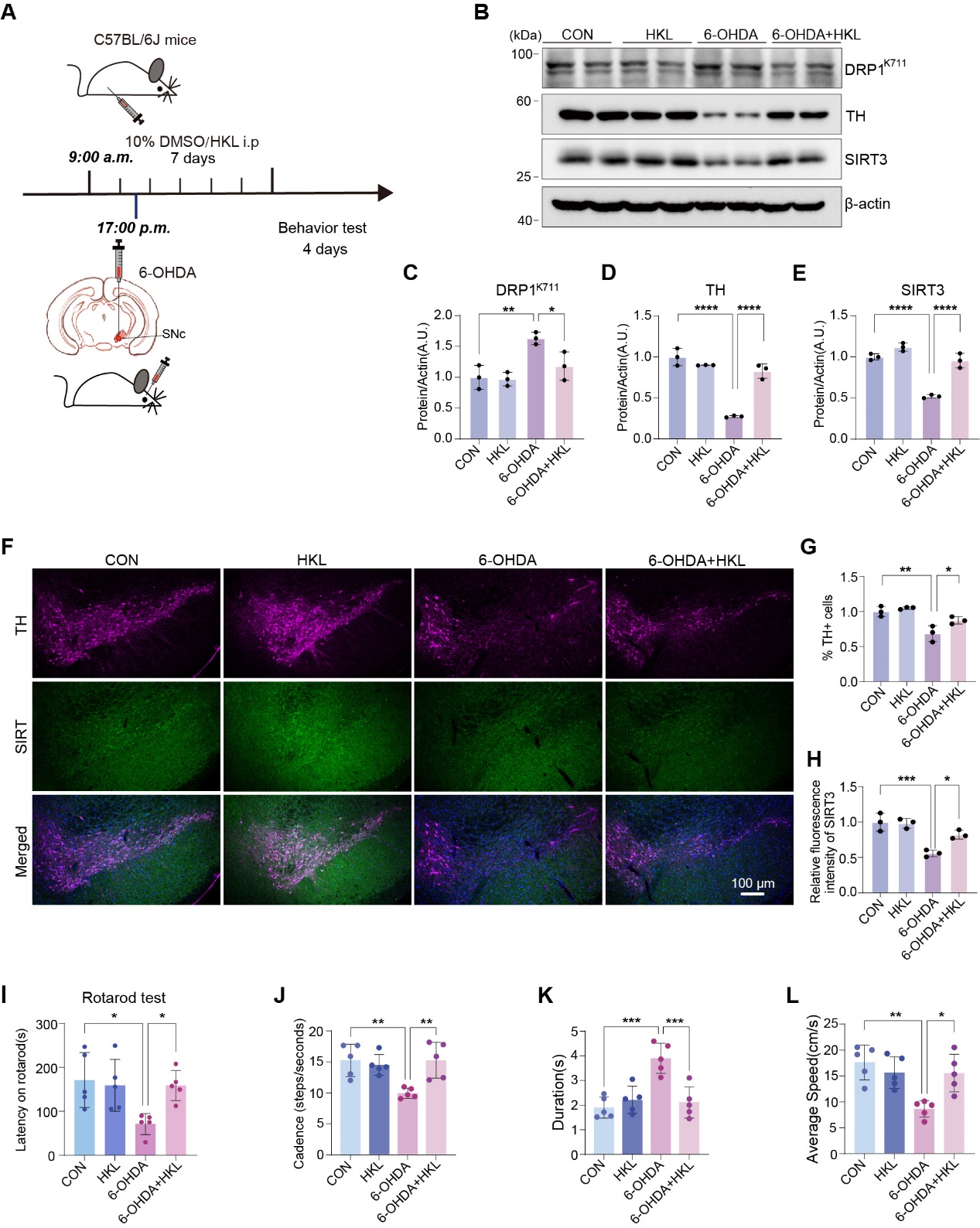


**Figure S11. SIRT3 agonist partially protects DA neurons from death in a 6-OHDA-induced PD animal model.** **(A)** Experimental timeline for the administration of HKL and 6-OHDA to establish a PD mouse model and subsequent behavioral testing. **(B–E)** Western blotting analysis of DRP1^K711^, TH, and SIRT3 levels in the substantia nigra pars compacta (SNc) brain tissue samples of 6-OHDA-induced PD model mice treated with the SIRT3 agonist, HKL. Data are represented as the mean ± SD (n = 3). **P* < 0.05, ***P* < 0.01, and *****P* < 0.0001 *vs.* indicated group. **(F–H)** Immunofluorescence staining and analysis of TH and SIRT3 expression in the SNc region of 6-OHDA-induced PD model mice treated with HKL. Data are represented as the mean ± SD (n = 3). **P* < 0.05, ***P* < 0.01, and ****P* < 0.001 *vs.* indicated group; *ns*, not signiﬁcant. **(I**) Rotarod test of motor function in mice with 6-OHDA-induced PD treated with HKL. Data are represented as the mean ± SD (n = 5). **P* < 0.05 *vs.* indicated group. **(J–L)** Catwalk gait analysis of cadence, duration, and average speed in HKL-treated 6-OHDA-induced PD mice. Data are represented as the mean ± SD (n = 5). **P* < 0.05 and ***P* < 0.01 *vs.* indicated group. Statistical analysis results are presented in Table S1.


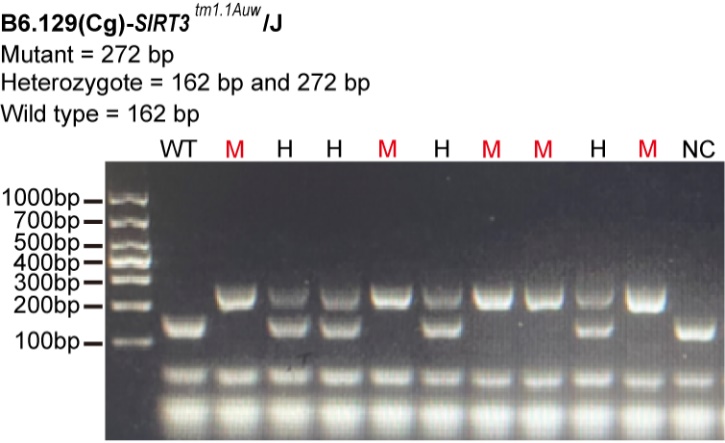


**Figure S12. Identification of mouse genes and homologous sequence comparison of DRP1 in mice and humans.** Genotyping results confirming the presence of the targeted gene in mice.


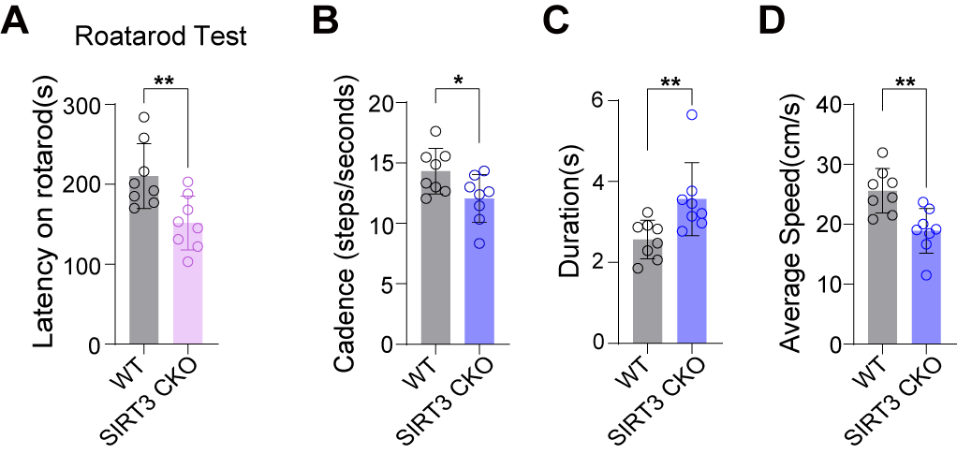


**Figure S13. Motor deficits in mice with conditional knockout (CKO) of *SIRT3* in the SNc region. (A)** Rotarod test of motor function in *SIRT3* CKO mice. Data are represented as the mean ± SD (n = 8). ***P* < 0.01 *vs.* indicated group. **(B–D)** Catwalk gait analysis of cadence, duration, and average speed in *SIRT3* CKO mice. Data are represented as the mean ± SD (n = 8). **P* < 0.05 and ***P* < 0.01 *vs.* indicated group. Statistical analysis results are presented in Table S1.


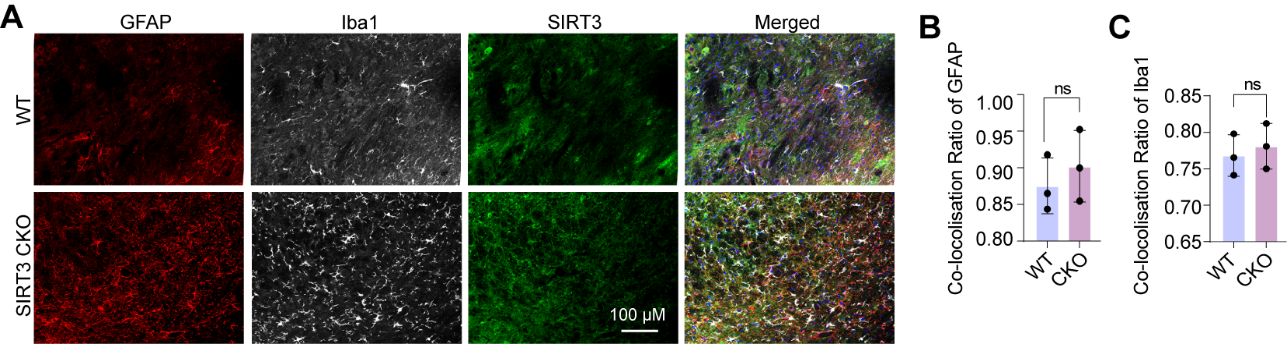


**Figure S14. No significant variations in SIRT3 expression levels in the astrocytes and microglia of *SIRT3* CKO mice. (A–C)** Immunofluorescence staining and analysis of the levels of glial fibrillary acidic protein (GFAP), ionized calcium-binding adaptor molecule 1 (Iba1), SIRT3, and co-labeling of SIRT3 expression in the SNc region of *SIRT3* CKO mice. Data are represented as the mean ± SD (n = 3). *ns*, not significant. Statistical analysis results are presented in Table S1.
